# The neuronal protein Neuroligin 1 promotes colorectal cancer progression by modulating the APC/β-catenin pathway

**DOI:** 10.1186/s13046-022-02465-4

**Published:** 2022-09-02

**Authors:** Margherita Pergolizzi, Laura Bizzozero, Federica Maione, Elena Maldi, Claudio Isella, Marco Macagno, Elisa Mariella, Alberto Bardelli, Enzo Medico, Caterina Marchiò, Guido Serini, Federica Di Nicolantonio, Federico Bussolino, Marco Arese

**Affiliations:** 1 Department of Oncology, University of Torino, 10060 Candiolo, Italy; 2 Candiolo Cancer Institute, FPO-IRCCS, 10060 Candiolo, Italy; 3 Pathology Unit, Candiolo Cancer Institute, FPO-IRCCS, 10060 Candiolo, Italy; 4 Department of Medical Sciences, University of Torino, 10124 Torino, Italy

**Keywords:** Neuroligin 1, tumor budding, intravasation/extravasation, APC, metastasis, WNT pathway

## Abstract

**Background:**

Colorectal cancer (CRC) remains largely incurable when diagnosed at the metastatic stage. Despite some advances in precision medicine for this disease in recent years, new molecular targets, as well as prognostic/predictive markers, are highly needed. Neuroligin 1 (NLGN1) is a transmembrane protein that interacts at the synapse with the tumor suppressor adenomatous polyposis Coli (APC), which is heavily involved in the pathogenesis of CRC and is a key player in the WNT/β-catenin pathway.

**Methods:**

After performing expression studies of NLGN1 on human CRC samples, in this paper we used in vitro and in vivo approaches to study CRC cells extravasation and metastasis formation capabilities. At the molecular level, the functional link between APC and NLGN1 in the cancer context was studied.

**Results:**

Here we show that NLGN1 is expressed in human colorectal tumors, including clusters of aggressive migrating (budding) single tumor cells and vascular emboli. We found that NLGN1 promotes CRC cells crossing of an endothelial monolayer (i.e. Trans-Endothelial Migration or TEM) in vitro, as well as cell extravasation/lung invasion and differential organ metastatization in two mouse models. Mechanistically, NLGN1 promotes APC localization to the cell membrane and co-immunoprecipitates with some isoforms of this protein stimulates β-catenin translocation to the nucleus, upregulates mesenchymal markers and WNT target genes and induces an “EMT phenotype” in CRC cell lines

**Conclusions:**

In conclusion, we have uncovered a novel modulator of CRC aggressiveness which impacts on a critical pathogenetic pathway of this disease, and may represent a novel therapeutic target, with the added benefit of carrying over substantial knowledge from the neurobiology field.

**Supplementary Information:**

The online version contains supplementary material available at 10.1186/s13046-022-02465-4.

## Background

Different proteins that are used in a controlled manner in neurons are unleashed by malignant transformation. These neuronal markers are part of various families: nuclear, cytosolic, membrane and vesicular proteins, neurotrophic factors and their receptors, developmental antigens, guidance proteins (reviewed in [1, 2]). In the case of purely synaptic proteins, such as Neuroligins (NLGNs), the knowledge on their involvement in tumor biology is limited to their prognostic value [3].

The family of NLGNs is composed of transmembrane post-synaptic proteins of the CNS that function in the fine-tuning of the synaptic activity. We have previously shown that a member of this family, Neuroligin 1 (NLGN1), is expressed in endothelial cells and modulates angiogenesis [4–7]. Colorectal cancer (CRC) is a multifaceted and highly heterogeneous disease both at the inter-patient and intra-tumoral levels [8]. While a few basic pathway alterations have been identified and serve as predictive markers of resistance to therapy [9], prognostic markers in CRC are largely missing [10].

Despite the use of chemotherapy, targeted therapy and immunotherapy, the prognosis for metastatic CRC patients remains dismal. Indeed, the spread of most tumors, including CRC, is the culprit for basically all cancer-related deaths, and the discovery of actionable targets in this process is highly needed. The metastatization process is guided by a long and incompletely understood sequence of events. We can postulate that such sequence starts at the so-called invasive front in the primary tumor where tumor budding, i.e. the detachment of single or small groups of cells (up to 5) from the tumor mass can represent a step towards lymphatic and hematogenous invasion [11, 12]. Tumor budding is today broadly presented in standardized CRC pathology reports, and its role as a risk factor of metastatic disease is widely accepted. Moreover, several efforts are being made to sub-categorize budding itself, to better stratify metastatic risk [13]. Mechanistically, the process may represent the observable correlate of an epithelial-mesenchymal transition (EMT)-related process in the tumor microenvironment of CRC [14, 15], as well as the first visible connection between the primary tumor mass and the final establishment of metastatic disease.

Intravasation is another complicated process that follows tumor budding in time. It requires the crossing of the endothelial monolayer and appears to be dependent on microvessel density and diameter of the vasculature, as well as the presence of associated cells (platelets, fibroblasts, macrophages, neutrophils) that play a role in promoting tumor cell intravasation. Molecularly, TGFβ, EGF, uPA/uPAR, MMPs, integrins, all appear to influence intravasation [16]. Hereafter, tumor cells enter circulation as either single cells or aggregates (that are referred to as ‘emboli’ or ‘microemboli’). Studies dating back to the 1970s have suggested that out of thousands of cells that enter the bloodstream, only a few develop into metastases [17] and that cells die within 1–2 days in circulation [18]. This is explained by the severe stress to which cells are subject: loss of adhesion, hemodynamic shear forces, and attacks of the immune system [19].

The ability to form a metastasis still depends upon exiting the vasculature (extravasation) and colonization of distant organs. Extravasation requires occlusion in a small capillary or bifurcation, or rolling adhesion to the endothelium in a larger vessel, alterations in the endothelial barrier with breakage of the inter-endothelial cell-cell junctions, and transendothelial migration to colonize the organ [19].

In the last part of the process tumor cells colonize the host organ. This is again a very inefficient series of events: infiltration, evasion of immune defenses, adapting to the new environment, and finally, growing to substitute the target tissue [20]. Overall, the metastatic process appears to be impaired by many hurdles, yet once established, it is seldom curable.

As mentioned above, one of the early events in the metastatic process is EMT. During EMT, polarized epithelial cells transform into migratory mesenchymal cells with invasive properties. A key modulator of EMT is the WNT pathway, which induces the expression of EMT genes. This pathway is extraordinarily complex and can be classified as canonical and non-canonical. In the canonical pathway, activated WNT signaling inhibits the degradation of β-catenin (β-cat) which can translocate from the cytoplasm to the nucleus and regulate transcription of many genes. The WNT proteins family activate surface receptors such as the 7 transmembrane FZD (frizzled) proteins and the LRP5/6 (low-density lipoprotein receptor). Upon this activation, Dishevelled (DVL), a scaffolding protein required for the stabilization of β-cat, is activated, causing the recruitment of the so-called “destruction complex” (constituted by Axin, GSK-3 β, CK1, Adenomatous polyposis coli -APC) to the membrane receptors. GSK-3 β is now incapable of phosphorylating β-cat which migrates from cytosol to the nucleus, there interacting with T cell-specific factor (TCF)/lymphoid enhancer-binding factor (LEF) to turn on the WNT target genes such as c-Myc, cyclin D1, and Cdkn1a [21].

Among the members of the destruction complex, APC is regarded as a CRC tumor suppressor that can be mutated both in the germline and at the somatic level. Germ-line mutations in the APC gene result in familial adenomatous polyposis (FAP), which is characterized by numerous polyps in the intestines, but mutations in APC have been also found in up to ~80% of sporadic carcinomas and adenomas [22]. One of the effects of APC loss of function through mutation is the destabilization of the destruction complex with consequent translocation of β-cat to the nucleus [22].

Because of its pervasive activity, the interest in WNT signaling has remained high since the discovery of the first mammalian WNT proto-oncogene almost 40 years ago. In the meantime, a plethora of drugs targeting WNT downstream effectors has been produced, unfortunately without clear-cut success in the clinic to date [23].

In this paper, we investigated the hypothesis that NLGN1 could mediate the aggressive/invasive behavior of CRC cells and increase the overall metastatization capacity. We studied its mechanism of action by evaluating its ability to affect the WNT/APC/β-cat pathway. Results show that NLGN1 is expressed by cancer cells of the primary tumor, by migrating “budding” tumoral cells, and by intravasated tumor emboli in human CRC samples. Moreover, NLGN1 promotes trans-endothelial migration in vitro, in vivo lung colonization, and metastatization in animal models, it induces β-cat translocation to the nucleus, and β-cat target gene expression.

## Materials and methods

### An expanded version of the material and methods is available as supplementary material

#### Animal studies

Non-obese diabetic/severe combined immunodeficiency (NOD/SCID) mice, purchased from Charles River and mice lacking NLGN1 (NLGN1 ^- /-^) and NLGN2 (NLGN2 ^- /-^) (respectively Nlgn1tm1Bros/J Nlgn2tm1Bros/J, purchased from Jackson Laboratory, Bar Harbor, ME, USA), previously described in [24] backcrossed in our laboratory to a C57BL/6 background, were maintained in the animal facility of our Institute under standard housing conditions. Male or female 6 weeks old mice were used for experimental procedures. Investigation was conducted in accordance with the ethical standards and according to national and international guidelines. In vivo studies were performed within an authorized animal facility (15/2016-UT), upon notification of the experiments to the Ministry of Health (authorization No.24/2019-PR), and in accordance with DLGS 26/2014 and EU directive 2010/63.

#### Human tissue sample origin and immunohistochemistry

NLGN1 expression was evaluated by immunohistochemistry (IHC) on a colorectal carcinoma tissue microarray (TMA) (CRM1505, US Biomax, Inc., Rockville, MD, USA) and on 16 colon cancer cases retrieved from internal pathology archives. Colon cancer TMA was composed of 150 cores, 1 core/case. Relevant information about each case (age, sex, histotype, stage, TNM, grade) were available on line at https://www.biomax.us/. A series of 16 cases with stage III or IV colorectal cancer with high grade budding and / or lymphovascular embolization was selected in the files of the Pathology Unit. The most representative section per case was chosen and used to perform immunohistochemistry. All patient included had previously signed an informed consent (“Profiling” protocol 001-IRCC-00IIS-10, approved by the Ethical Committee of Fondazione Piemontese per l’Oncologia Istituto di Ricerca e Cura a Carattere Scientifico of Candiolo). Staging information were available for all patients.

Immunostainings were performed using an automated immunostainer (Ventana BenchMark Ultra Auto-Stainer, Roche Ltd). Endogenous peroxidase activity was blocked by 20 min of incubation with 0.3% hydrogen peroxidase. Slides were tested using anti-NLGN1 antibody (1:100 Anti-NLGN1 Mouse monoclonal Neuromab, clone N97A/31, from ORIGENE). The antibody had been tested for specificity on NLGN1 null brains (Supplementary Fig. S1) and cross-reacts with the mouse isoform. Sections were tested with a streptavidin-biotin-peroxidase kit (UltraVision Large Volume Detection System Anti-Polyvalent, HRP, LabVision, Freemont, CA, USA), and after incubation the reaction product was detected using diaminobenzidine (DAB). Finally, the sections were counterstained with Mayer’s Haematoxylin, and mounted with aqueous mounting medium. NLGN1 expression was scored as present at different degrees of intensity (score 1, score 2, score 3, respectively) or absent (0). Percentage of tumor cell positive for the biomarker was not taken into account since we observed that homogeneous staining was consistently present across samples.

#### Gene expression analysis

Gene expression data for CRC primary tumors and cell lines were downloaded from Colorectal Adenocarcinoma (TCGA, PanCancer Atlas) dataset within cBioportal (https://www.cbioportal.org/), and from series GSE59857 (https://www.ncbi.nlm.nih.gov/geo/query/acc.cgi?acc=GSE59857) respectively. To select samples harbouring a high level of expression of NGLN1 we evaluated the Zscore distribution, selecting cases with values above 1.6 (*p* value < 0.05). Survival analysis was carried out within cBioportal according to standard procedure to generate Kaplan Meier, and log rank test to estimate significance. Pearson correlation analysis for NLGN1/WNT pathway was performed with R and statistical tests were moderated for Bonferroni multi-test hypothesis.

#### Automated in vitro Trans Endothelial migration assay

Human umbilical vein Endothelial cells (HUVECs) were extracted from umbilical cords and cultured in 20% FBS M199 medium (Sigma-Aldrich, St. Louis, MO, USA). In all experiments, HUVECs were used between passages 2 and 5. To overcome the intrinsic biological variability of HUVECs, all experiments were performed with a mixture of HUVECs from at least three different umbilical cords. Real-time cell analysis (RTCA) of cancer cells invasion on an endothelial monolayer was monitored using a E-plate 16 and xCELLigence DP System (Acea Bioscience, Inc., San Diego, CA, USA) according to [25]. To each well, of background calibrated E-plate, 100 μl of HUVECs cell suspension (2.5 × 10^6^ cells/ml) were grown in 0,1% gelatin for 18-21 hours until they form a stable monolayer as evidenced by a flattening of cell index. Then HUVEC media was replaced by 100 μl of tumor cells (1 × 10^5^ cells/ml) resuspended in media used to grow tumor cells. Invasion as a drop in cell index was monitored in realtime over the next 6-12 hours reading impedance at 15 minute intervals.

#### In vivo Tail vein injection, cecum orthotopic implantation and IVIS Imaging

In the tail vein experiments 6 week-old female mice were placed into a restrainer and colorectal cancer cells (1.5 × 10^6^cells in 100 uL DPBS per mouse) are SLOWLY injected (2-3 minutes) via i.v. through the tail vein using a 26 × 1/2” gauge needle. To monitor the formation of lung colonies through time, once a week for 1 month bioluminescent acquisitions images were collected by means of the IVIS® Lumina II imaging system, after subcutaneous injection of luciferine (15 mg/ml) (PerkinElmer, Waltham, MA, USA). After 1 month from cells injection, mice were sacrificed immediately after luciferine injection and lung explants were visualized by IVIS® Lumina II to detect in vivo CRC cells lung colonization ability. Following IVIS imaging, lungs were formalin fixed, embedded in paraffin and processed for eventual histological analysis. IVIS Images were processed using Life Science's Living Image software and the captured signal was quantified as Total flux (p/s) or number of metastatic foci.

In the cecum orthotopic implantation 6 week-old female or male mice were treated subcutaneously with pre-operative analgesia (0,5% Meloxicam 5 mg/kg) and with antibiotic (2,5% Enrofloxacin 10 mg/kg). During all the surgery mice were anesthetized by using 2,5 isoflurane inhalation with 3-5 L/min airflow. The abdominal region was disinfected and cleaned several times with 70% ethanol and a 1.0–1.5 cm incision along the linea alba was made. Once the cecum was exposed CRC cell lines (1.5 × 10^6^ cells/mouse) were micro-injected into the cecum submucosa using a glass micro-capillary. One week later, mice were injected with luciferin (15 mg/ml) (PerkinElmer, Waltham, MA, USA) and their in vivo tumor bioluminescence was analyzed using IVIS® Lumina II imaging system (Perkin Elmer). Animals were stratified into homogeneous groups based on bioluminescence signal. At the end point of the experiments, mice were injected with luciferin (15 mg/ml) (PerkinElmer, Waltham, MA, USA), sacrificed, and their organs extracted for metastasis formation using IVIS® Lumina II imaging system. Following IVIS imaging, organs were formalin fixed, and embedded in paraffin for eventual histological analysis. IVIS Images were processed using Life Science's Living Image software and the captured signal was quantified as Total flux (p/s).

#### Statistical analyses

Graphpad Prism versions 5 were used as indicated in figure legends. Results are expressed as mean ± S.E. when derived from averaged experiments (n represents the number of individual experiments), or mean ± S.D. when derived from several data points of one experiment. . The asterisks (*, **, and ***) in figure panels refer to statistical probabilities (*p*) of <0.05, <0.01, and <0.001, respectively.

## Results

### NLGN1 expression in colorectal cancer

**Fig 1:**
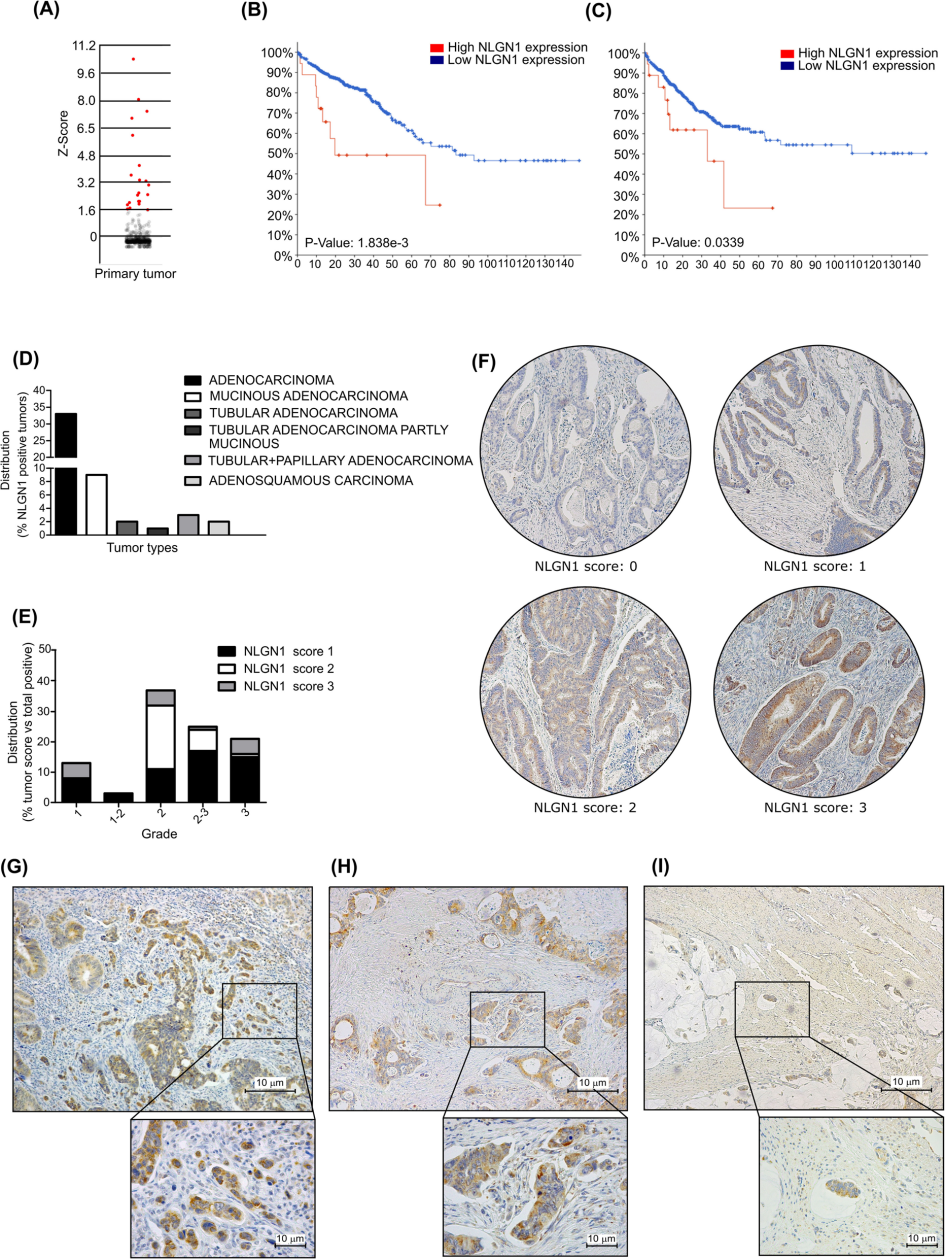
NLGN1 expression in human colorectal cancer. **A** Tatsuky plot displaying the Z-Score distribution of NLGN1 in CRC primary tumors (TCGA, PanCancer Atlas). Red dots indicate the samples with Z-Scores above 1.6. **B, C** Kaplan-Maier plots showing that higher NLGN1 expression predicts worse overall survival (**B**) and disease free survival (**C**) in CRC patients from cBioportal. *P* value was calculated as logrank test. **D-F** IHC was performed to evaluate NLGN1 expression on a TMA of colorectal cancer (CRM1505 - Biomax), which contains the following specimens: 88 cases of adenocarcinoma, 29 mucinous adenocarcinoma, 10 tubular adenocarcinoma, 4 tubular adenocarcinoma partly mucinous, 6 tubular+papillary adenocarcinoma and 4 adenosquamous adenocarcinoma, single cores per case. **D** Distribution of NLGN1 protein expression according to the IHC positivity (present or absent) in terms of pathology diagnosis. NLGN1 is mainly expressed in human adenocarcinoma. **E.** Distribution of NLGN1 protein expression among cores from human adenocarcinoma according to the IHC score, as present (1, 2, 3) or absent (0). NLGN1 is expressed at a higher percentage and intensity in cores from tumor grade 2. **F** Representative images of NLGN1 protein expression by IHC in CRC TMA cores and their relative score (original magnification 10X). **G-I** Representative images of NLGN1 expression in human CRC from a patient case study available in our institute. Immunohistochemistry reactions with antibodies anti-NLGN1 showed intense, diffuse positivity in a subset of colon adenocarcinoma. Moreover, NLGN1 positivity was even stronger in single-cell high grade budding (**G**) and in vascular neoplastic emboli (**H-I**). Scale bar: 10 μm

We initially analyzed the expression of NLGN1 in a public CRC gene expression database (TCGA, PanCancer Atlas- Colorectal Adenocarcinoma) through the cBioportal platform [26, 27]. High NLGN1 expression was present in a small percentage of samples (3.5%, z-score threshold 1.6) (Fig. 1A). Survival analysis to generate Kaplan Meier, and log rank test to estimate significance, demonstrated that NLGN1 overexpressing patients had a significant reduction in both overall survival and progression free survival (p-values: 1.838e-3 and 0.0339 respectively, Fig. 1B, C). We thus investigated the expression of NLGN1 in CRC at the protein level by immunohistochemistry (IHC) on tissue microarrays (TMA) and on a subset of colon cancers available in our Institution. The specificity of the used antibody (Neuromab -clone N97A/31) for NLGN1 was verified on brains of NLGN1 and NLGN2 -null mice (Supplementary Fig. S1). The analysis on TMA (Fig. 1D-F) showed that NLGN1 was expressed by tumor cells as follow: about half of the TMA’s cores (75, 52%) were positive for NLGN1: 5 with score 3 (3,3%), 21 with score 2 (14%) and 49 with score 1 (32,7%). Sixty-nine cores were negative (48%) and 5 were not assessable. Histograms in Fig. 1D and E present respectively the percentage of NLGN1 positive cases for each tumor subtype and the distribution of NLGN1 positive cases among different grades of the adenocarcinoma subset (with grade 2 as the most represented).

We next focused on the possibility that NLGN1 was expressed by the most infiltrative tumor cells by analyzing the invasive front of the tumor (tumor cell budding) and tumor emboli. Immunohistochemical evaluation of neoplastic lymphovascular invasion /embolism is a difficult task since emboli can be present in one histological section but disappear in the subsequent one, even if sub-seriated. To bypass this obstacle, we selected a subset of 16 CRC cases available at our institute that were described in the pathology report as positive for lymphovascular invasion. Of these 16 cases, 6 showed NLGN1 score 3 positivity, 5 were score 2 and 3 were score 1, while only 2 cases were NLGN1 negative. These numbers, which come from a subset of aggressive tumors, globally represent a much higher level of NLGN1 expression/positivity than that resulting from the TMA analysis discussed above. We then concentrated on NLGN1expression in high grade tumor budding single cells, assuming that those cells would be the same to penetrate the lymphovascular vessel and embolize. The overall analysis showed that NLGN1 positive tumors showed systematically strong staining in high grade tumor budding single cells (Fig. 1G) and lymphovascular emboli (Fig. 1H, I), proving our initial hypothesis.

### NLGN1 promotes crossing of an endothelial monolayer in vitro

**Fig 2:**
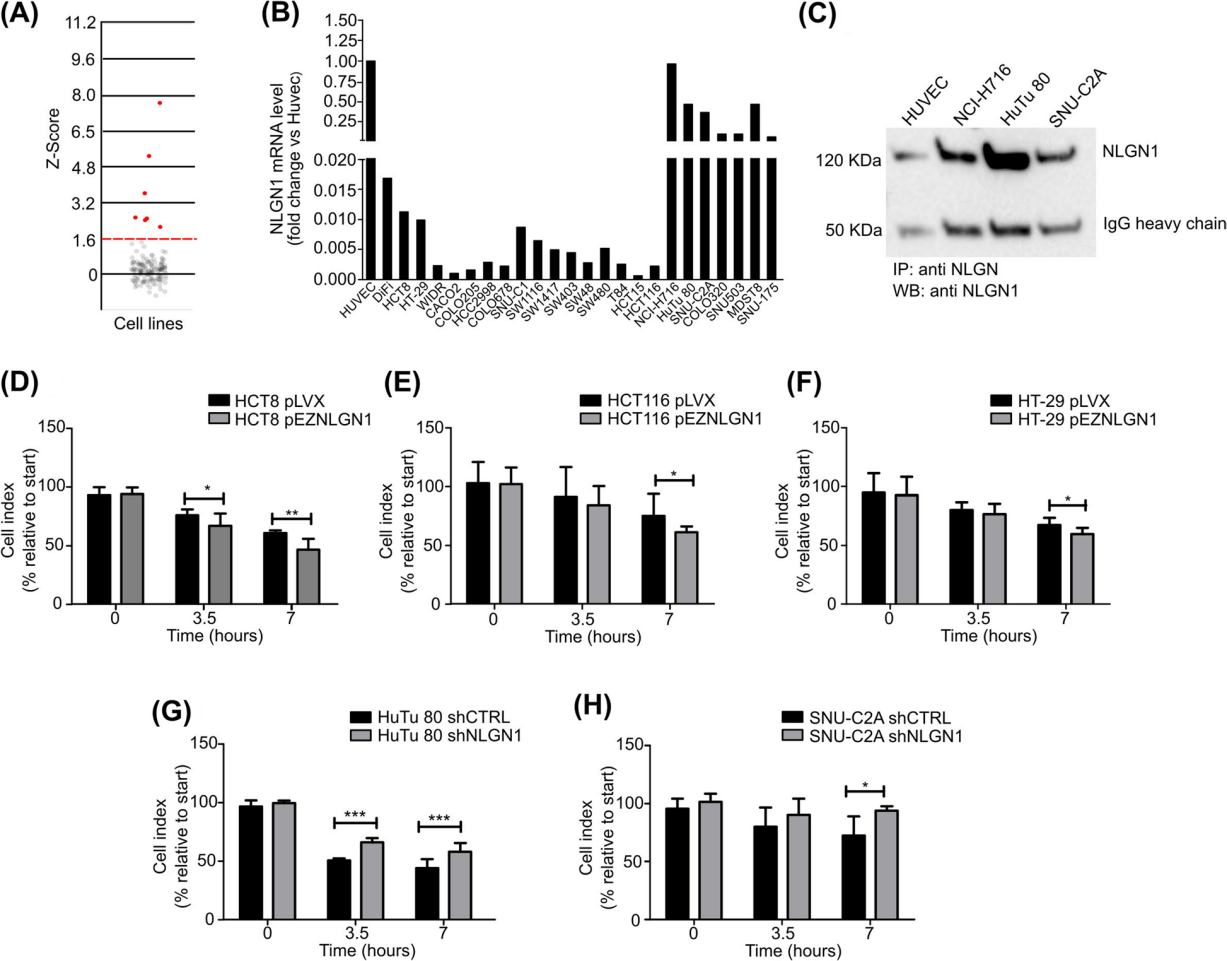
NLGN1 promotes transendothelial migration in vitro **A** Tatsuky plot displaying the Z-Score distribution of NLGN1 in CRC cell lines (dataset GSE59857). Red dots indicate the samples with Z-Scores above 1.6, *p* <0.05. **B** qRTPCR of NLGN1 expression level in CRC cell lines selected from the cell bank available in our institute. Fold-change is calculated with respect to HUVEC cells, known to express high endogenous levels of the protein. **C** Immunoprecipitation assay was performed on HUVEC, NCI-H716, HuTu 80 and SNU-C2A cells, using an antiNLGN (L067) antibody and immunoblotting was conducted with a monoclonal antibody (4C12) able to recognize NLGN1. The band detectable at 120-kDa corresponds to NLGN1. **D-H** Transendothelial migration of human CRC cell line in which NLGN1 is overexpressed, HCT8 (**D**), HCT116 (**E**) and HT-29 (**F**), or silenced, HuTu 80 (**G**) and SNU-C2A (**H**). Cell migration was recorded in real time through the X-Celligence System for 12 hour. Histograms show the cell index in terms of percentage relative to start, at the indicated time points. A lower cell index in this representation means an increased capacity of trans-endothelial crossing. Values are expressed as mean ± SD (*n*=3 independent experiments performed in triplicate). Two-way ANOVA with Bonferroni test: * *p* < 0.05, ** *p* < 0.01, *** *p* < 0.001

In order to dissect the cellular activities of NLGN1 we initially explored its expression in a database of gene expression profiles of our institutional CRC cell line collection (N=151) [28]. The analysis, through a c-bioportal interface [26, 27], showed that NLGN1 expression is very low or absent in almost all cell lines, except in 7 of these (NCI-H716, MDST8, SNU-C2A, COLO320DM, HuTu 80, SNU-175, SNU-503) that we labelled as “NLGN1 upregulated CRC cells” (Fig. 2A).

To confirm these data, we performed a qRT-PCR on cell lines from the database, thus isolating two groups of cells for our purposes: one in which NLGN1 was very low or absent (“NLGN1 null”) and another constituted by the “NLGN1 upregulated” mentioned above (Fig. 2B). Among the NLGN1 upregulated we chose three cell lines (HuTu 80, SNU-C2A and NCI-H716) for follow-up experiments and tested their NLGN1 protein expression by immunoprecipitation/western blot (Fig. 2C). Similarly, among the NLGN1 null cells we chose the cell lines (HCT8, HT-29 and HCT116) for the prosecution of the experiments. The identity of all cell lines was verified through the Cell ID system (Supplementary Table S1), while Supplementary Fig. S2 presents the data relative to NLGN1 expression modulation in all cell models.

Prompted by the presence of NLGN1-positive cells in tumor emboli (Fig. 1H, I) and given that crossing the endothelial monolayer is a crucial event for both intravasation and extravasation during metastatization, we evaluated the ability of NLGN1 to promote the process of trans-endothelial migration (TEM). Fig. 2D-F shows that three NLGN1 null cell lines -HT-29, HCT116, HCT8-, crossed the EC barrier with much higher efficiency when artificially overexpressing NLGN1. Conversely, two NLGN1 upregulated cell lines, SNU-C2A and HuTu 80, were impaired to cross the barrier when NLGN1 was silenced (Fig. 2G, H). To verify the specificity of the NLGN1 effects a “rescue” experiment was performed on HuTu 80 and SNU-C2A. Results show that overexpression of mouse NLGN1 (not targeted by the shRNAs) completely reversed the effects of NLGN1 downregulation (Supplementary Fig. S3).

These TEM data were obtained through an automated system (Xcelligence **)** that evaluates the integrity of the HUVEC monolayer by measuring its impedance in real time as described [25]. While the effects of NLGN1 on TEM were specific, reproducible and statistically significant, their magnitude was not very large (Fig 2D-H). Hence, to corroborate these data we reproduced the TEM assay with the same architecture (a HUVEC monolayer was formed on matrigel and next challenged with tumor cells) but performed it in a culture well, and manually counted tumoral cells adhering, interacting and breaking the HUVEC monolayer. Results showed that NLGN1 increased TEM for HuTu 80, SNU-C2A, HCT116, HCT8 and HT-29 cells with a significantly larger amplitude than the readings of the Xcelligence system (Supplementary Fig. S4). Our interpretation of this result is that in the impedance based assay, tumoral cells do destroy the integrity of the HUVEC monolayer, initially lowering the impedance, but next tend to increase the impedance once they adhere to the plate [25] , blunting the overall magnitude of the effect.

To further characterize the biological activity of NLGN1 on tumor cells , in a parallel set of experiments we evaluated if NLGN1 affected the proliferation of CRC cells. Results (Supplementary Fig. S2, panels C, G, K, O, S) show how in HT-29 and HCT116 cells, NLGN1 overexpression reduced proliferation, in HCT8 and SNU-C2A no effects of NLGN1 were visible, while only in HuTu 80, NLGN1 appeared to slightly promote proliferation. Overall, the effects of NLGN1 on proliferation appeared inconsistent and heavily cell-line-dependent. This situation completely reflects the in vivo subcutaneous tumor growth experiments that we performed with the different cell lines (Supplementary Fig. S2, panels D, L, P, T).

Globally, the only consistent effect of NLGN1 on tumor cells was to stimulate their ability to cross the endothelial barrier in all cell lines used.

### NLGN1 promotes lung invasion in the tail vein colonization assay and increased metastatization in the CRC orthotopic mouse model

**Fig 3:**
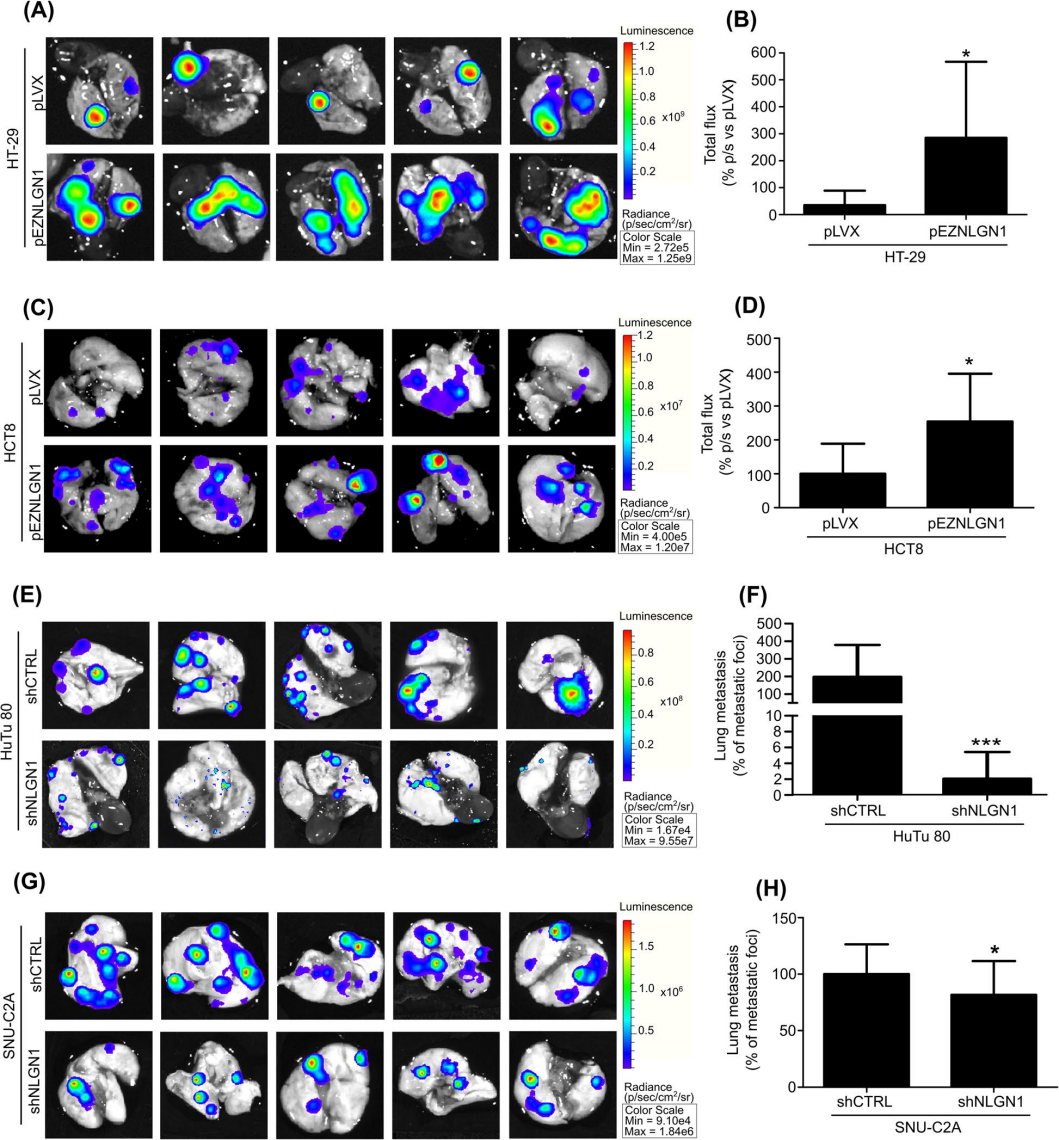
NLGN1 modulates lung metastasis outgrowth in a murine tail vein metastasis assay. 1,5x10^6^ cells (A-D) HT-29 or HCT8 cells, pLVX and pEZNLGN1, and (E-H) HuTu 80 or SNU-C2A cells, shCTRL and shNLGN1, further infected with a CMV-Luc vector were intravenously inoculated into the tail of 6 weeks old NOD/SCID mice. After 4 weeks mice were subcutaneously inoculated with 15 mg/ml luciferine 5 minutes before the sacrifice and the lungs were surgically excised. Luciferine bioluminescence was recorded through IVIS Lumina II apparatus. Images shown in **A, C, E** and **G** are representative of 5 mice. Graphs in B and D show the bioluminescence of tumor cells as total flux while graphs in F and H show the luciferin bioluminescence as % of the area occupied by the metastatic foci respect the total organ area, measured on the explanted lungs. Values are expressed as mean ± SD, *n*=5 (**B** and **D**), *n*=12 (**F**), *n*=20 (**H**). Mann-Whitney test, two tailed: * *p* < 0.05 **, *p* < 0.01, *** *p* < 0.001

To expand on above in vitro results, which focused on a single step of the metastatization process, we examined the in vivo extravasation/colonization capability of CRC cells, through the so-called tail vein colonization assay [29]. We injected luciferase-infected cells in the lateral tail vein of NOD-SCID mice and followed lung colonization through the IVIS recording system. As can be seen from the luciferase signal in mouse lungs, the NLGN1 null cell lines HT-29 and HCT8, dramatically increase organ invasion upon NLGN1 exogenous expression (Fig. 3A-D). On the other hand, NLGN1 upregulated cells, HuTu 80 and SNU-C2A, reduced their invasive capacity when NLGN1 was silenced (Fig. 3E-H).

**Fig 4:**
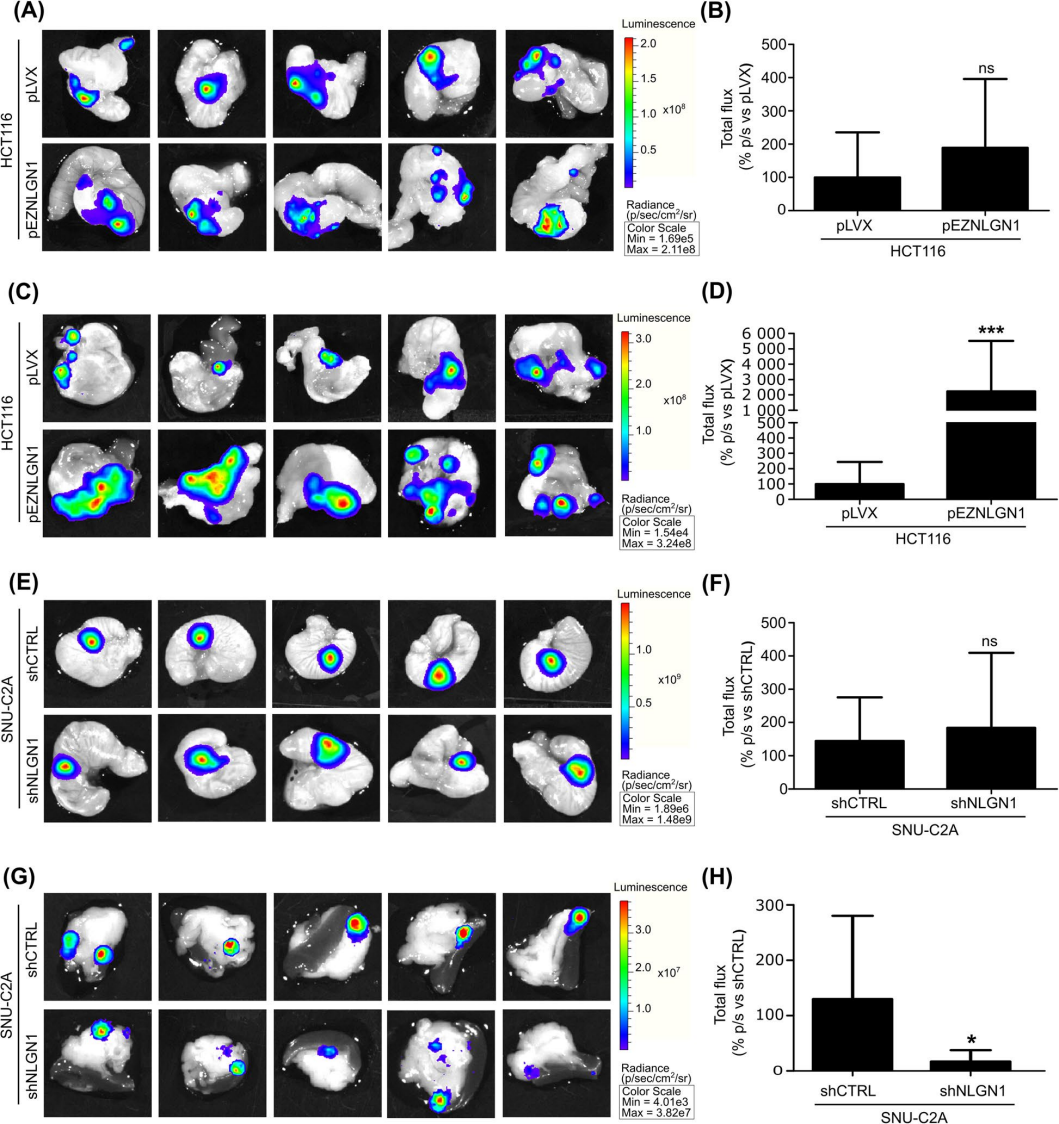
NLGN1 induces metastasis formation by CRC cells in vivo. 1,5 × 10^6^ cells (**A-D**) HCT116 pLVX and pEZNLGN1 cells and (**E-H**) SNU-C2A cells, further infected with a CMV-Luc vector were orthotopically inoculated into cecum of 6 weeks old NOD/SCID mice. After 8 weeks mice were subcutaneously inoculated with 15 mg/ml luciferine 5 minutes before the sacrifice and the cecum, intestin, stomach, spleen/pancreas, liver and lungs were surgically excised. Luciferine bioluminescence was recorded through IVIS Lumina II apparatus. Images of the primary tumor (cecum) in **A** and **E** andof the stomach (**C**) and spleen/pancreas (**G**) metastases are representative of 5 mice. Graphs in **B**, **D**, **F** and **H** show the bioluminescence of tumor cells as total flux. Values are expressed as mean ± SD, *n*=10. Mann-Whitney test, two tailed: * *p* < 0.05, *** *p* < 0.001

We next moved to a more comprehensive assessment of the metastatic capabilities of tumoral cells upon NLGN1 modulation through an orthotopic CRC implant model [30]. This model avoids the problem of primary tumor development in an exogenous environment, typical of xenografts, and better recapitulates the whole process of tumor progression: from growth to cell release from the primary tumor, to the formation of foci at distant sites. Cells were inoculated in the cecum and tumor growth was weekly monitored by in vivo bioluminescence. At week 4, after mice euthanasia, organs were explanted. A significant increase in stomach metastases (Fig. 4C, D) upon NLGN1 overexpression was recorded for the NLGN1 null HCT116 cells. On the other hand, the NLGN1 upregulated SNU-C2A cells reduced their metastatic capabilities in the spleen/pancreas upon NLGN1 silencing (Fig. 4G, H). Primary tumor growth (Fig. 4A, B, E, F), as well as metastatization to liver and lungs (Supplementary Fig. S5), was unaffected in all cases by NLGN1, indicating a site-specific activity in the metastatic promotion.

### NLGN1 expression affects the APC β-cat pathway.

When exploring the intracellular pathways on which NLGN1 could impact, we first considered that the tumor suppressor APC is required for localizing NLGN1 to neuronal nicotinic synapses [31], implicating a functional interaction at the cell membrane of both proteins. APC is a large multi-domain protein with a wide array of functions, and, along with its upstream regulator WNT, is heavily involved in the pathogenesis of CRC [22, 32]. Interestingly, when we explored the co-expression of NLGN1 with a series of proteins of the WNT pathway (Supplementary Table S2) in both the TCGA, PanCancer Atlas- Colorectal Adenocarcinoma dataset, and our internal cell line expression database [28] we found that CXXC4 (a negative regulator of the WNT signaling pathway via its interaction with DVL), and FZD1 (a 7-transmembrane domain protein that is a receptor for WNT ligands) were significantly correlated with NLGN1, potentially suggesting their cooperation at the membrane and β-cat destruction complex levels.

**Fig 5:**
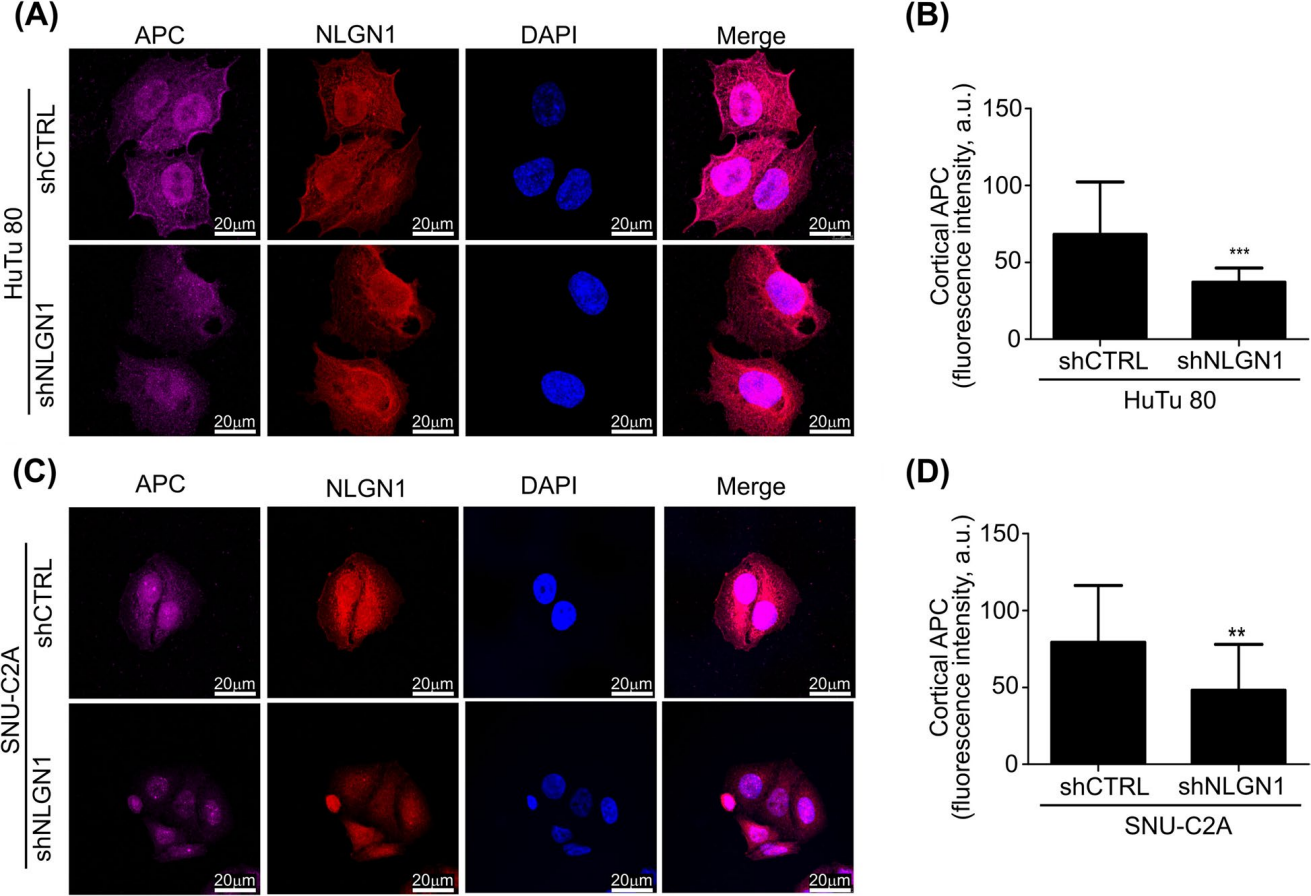
NLGN1 modulates APC localization at the plasma membrane. Confocal microscopy analysis of NLGN1 and APC co-staining in HuTu 80 (**A **and** B**), and SNU-C2A cells (**C **and** D**). **A **and** C** Cells were immunostained with anti APC (magenta) and anti NLGN1 (red) antibodies. Images shown are representative of 1 out of three reproducible experiments. Scale bar: 20 μm. The graphs in **B** and **D** show the quantification of APC signal at the plasma membrane using the imageJ Software and values are expressed as mean ± SD (HuTu 80: *n *= 17; SNU-C2A: *n *= 15). Mann-Whitney test, two tailed: ** *p* < 0.01

On this background, we set to explore the interaction of NLGN1 with the APC pathway. We tested by immunofluorescence whether NLGN1 could modulate APC localization at the plasma membrane. The results show that APC preferentially localizes at the plasma membrane in HuTu 80 and SNU-C2A,which carry a native form of APC (Supplementary Table S3) and express high endogenous levels of NLGN1, while NLGN1 downregulation releases APC from the cortical region (measured as described in Supplementary Fig. S6 ) into the cytoplasm (Fig. 5A-D). Conversely, “NLGN1 null” cells (HCT8, HCT116, HT-29) relocalize APC to cell membrane upon NLGN1 overexpression (Supplementary Fig. S7). It is important to note that HCT8 and HT-29 carry mutated forms of APC (Supplementary Table S3) that are nevertheless recognized by the anti APC antibody used in this experiment and raised against the N-terminus of APC (see discussion for comments on the role of mutant APC in NLGN1 activity).

In a separate set of experiments (Supplementary Fig. S8), we evaluated the existence of a physical interaction between APC and NLGN1 by co-immunoprecipitation experiments. Being APC such a large protein with different splice variants [33] and numerous mutated forms, the study of its pattern of appearance upon separation on a gel can be challenging, especially while analyzing its interaction with a new protein. Nevertheless, by immunoprecipitating NLGN1 from cells physiologically expressing the protein (the “NLGN1 upregulated” HuTu 80 and SNU-C2A cells), and blotting for APC, we obtained a pattern of APC bands highly similar to that visible when APC itself was immunoprecipitated and blotted, including the full length APC, as expected in cells carrying the native APC (Supplementary Fig. S8, panel B and C)*.* When we immunoprecipitated NLGN1 from the APC mutated, exogenous NLGN1 overexpressing HT-29 cells, we detected different APC bands (Supplementary Fig. S8 panel D) including one that could theoretically correspond to the product of the non-sense mutation E853*, present in HT-29 cells (Supplementary Table S3). We were not able to detect NLGN1 after immunoprecipitation of APC (i.e. in the “reverse direction”). In our experience, this is often the case because of different reasons, namely the fact that an epitope may be hidden in the complex NLGN1- APC*.* An extended study involving more antibodies targeting different APC regions would be needed to understand this point. We believe nevertheless that our experiments provide proof of physical interaction between NLGN1 and APC.

Obviously, other protein participants to the destruction complex or other modulators of the WNT pathway, could interact with NLGN1. To approach this issue we analyzed the membrane localization of CXXC4 which as described above, is significantly co-expressed with NLGN1, by confocal immunofluorescence (Supplementary Fig. S9). Results show that in HuTu 80, HT-29, HCT8 and HCT116 cells, NLGN1 promotes CXXC4 membrane cortical localization, indicating a potential functional interaction between these two proteins, similarly to that seen with APC and described above (Fig 5, and Supplementary Fig. S7)

**Fig 6:**
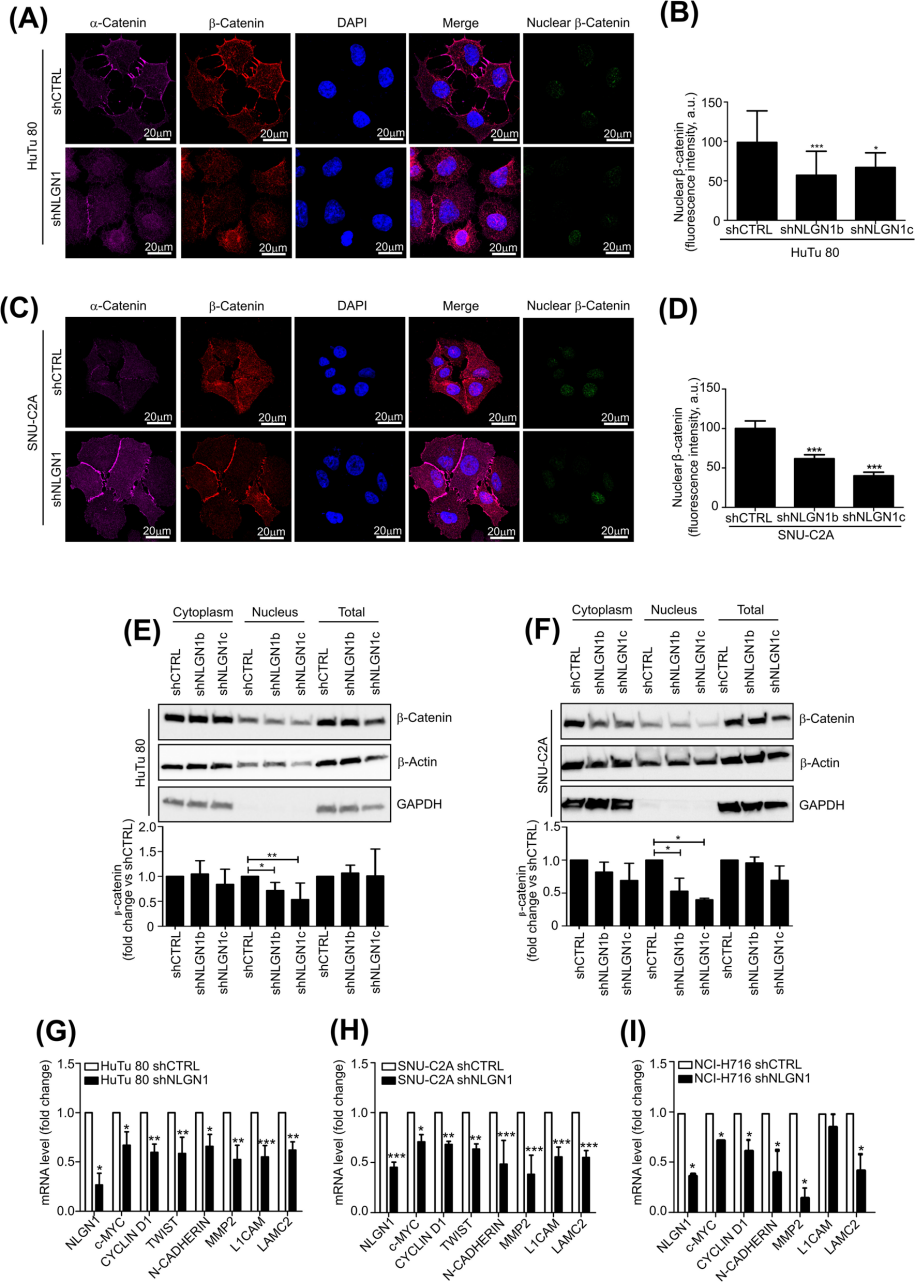
NLGN1 influences the APC/β-cat pathway. **A** and **C** Confocal microscopy of nuclear β -catenin into HuTu 80 (**A**) and SNU-C2A (**C**) cells. Cells were immunostained with anti α-catenin (magenta) and anti β-catenin (red) antibodies. Nuclear β-catenin was calculated as indicated in the methods section. The images in **A** and **C** are representative of 1 out of 3 reproducible experiments. Scale bar: 20 μm. The graphs in **B** and **D** show the fluorescent intensity of nuclear β-catenin and values are expressed as mean ± SD (HuTu 80: *n*=40; SNU-C2A: *n*=70). Mann-Whitney test: ** *p* < 0.01, *** *p* < 0.001. **E** and **F** Western blotting analysis of nuclear β-catenin into HuTu 80 (**E**) and SNU-C2A (**F**) control (shCTRL) or NLGN1-downregulated (shNLGN1b and c) cells. Cell lysates were fractionated using the NEPER nuclear and cytoplasmic extraction kit (ThermoFisher) Immunoblottings were carried out using antibodies specifically recognizing β-catenin (95 kDa), β -actin (b 45kDa), as housekeeping, and GAPDH (37 kDa), as a control of the purity of the fractions, and the images are representative of 1 out of 3 reproducible experiments. Graphs in **E** and **F** show the densitometry of nuclear, cytoplasmic and total β-catenin, normalized on β-actin used as housekeeping. Fold-change is calculated with respect to shCTRL cells and values are expressed as mean ± SE (*n* = 3 independent experiments). Kruskal-Wallis test with Dunn’s posttest: *, *p* < 0.05 **, *p* < 0.01. **G-I** qRT-PCR of NLGN1, c-MYC, Cyclin D2, Twist (undetectable in NCI-H716), N-Cadherin, MMP2, L1CAM and LAMC2 expression level in HuTu 80 (**G**), SNU-C2A (**H**) and NCI-H716 (**I**) control (shCTRL) or NLGN1-downregulated (shNLGN1) cells. Fold-change is calculated with respect to shCTRL cells for each gene and values are expressed as mean ± SE (*n* = 3 independent experiments). Mann-Whitney test, two tailed: *, *p* < 0.05 **, *p* < 0.01, ***, *p* < 0.001

As explained in the introduction, activation of the WNT pathway normally causes the recruitment of APC and the other components of the “destruction complex” to the plasma membrane and stabilization of β-cat, which then translocates to the nucleus, where it promotes transcription of the WNT target genes [34]. Hence, we next tested if NLGN1 modulated β-cat localization in the nucleus. Immunofluorescence (Fig. 6A-D) and subcellular fractionation (Fig. 6E, F), clearly showed that NLGN1 downregulation in HuTu 80 and SNU-C2A reduced nuclear β-cat. A “rescue” experiment verified the specificity of this effects for NLGN1 (Supplementary Fig. S10).

We next tested whether NLGN1 expression influenced the expression of WNT β-cat/TCF target genes including some commonly used markers of EMT. We demonstrated that c-MYC, Cyclin D1, Twist, N-Cadherin, MMP2, L1CAM, and LAMC2 expression levels in the NLGN1 upregulated cell lines HuTu 80, SNU-C2A and NCI-H716, decreased significantly upon NLGN1 silencing (Fig. 6G, H, I).

Prompted by the data on EMT markers being modulated by NLGN1, we performed a final set of experiments aimed at elaborating on the link between NLGN1 and EMT . Results showed that NLGN1 induced an EMT phenotype [35] (Supplementary Fig. S11) in HuTu 80 and HCT116 cells. This phenotype consisted of elongated spindle -like shaped cells, numerous isolated migrating cells, a different shape of the colonies in HCT116 cells, and in many instances the presence of evident actin stress fibers (a characteristic of mesenchymal-migrating cells, which are thought to mediate cell contractility in close cooperation with focal adhesions), as opposed to the epithelial -polygonal shape in cells devoid of NLGN1. EMT is highly relevant for TEM [36] and the mechanism by which NLGN1 induces TEM could rely on the activation of an EMT program, by which cancer cells adhere to the endothelium and cross the vessel wall, perhaps by forming pseudopodia and invadopodia, as it is the case , for example, for Vascular cell adhesion molecule 1 (VCAM1) [36]. EMT is also directly linked to tumor budding [15, 37] and the NLGN1-induced EMT phenotype could explain our observation of NLGN1-stained budding cells in human samples. Finally, NLGN1 promotes cell migration, a mesenchymal trait which is also linked to tumor budding [15], in HuTu 80 and HCT116 cells (Supplementary Fig. S11).

## Discussion

Our study of NLGN1, along with its partner Neurexin, started with its role in the vascular system, including in tumor angiogenesis [7] and subsequently, based on some preliminary observations , we considered the possible “tumor autonomous” role of NLGN1. In this paper we show that NLGN1 *a)* is expressed by CRC tumor cells in vitro and in vivo, including high grade tumor budding single cells and tumor vascular emboli *b)* promotes crossing of an endothelial monolayer in vitro *c)* promotes lung invasion in the tail vein colonization assay and metastatization in the CRC orthotopic mouse model d) anchors the tumor suppressor APC to the membrane, and physically interacts at least with some isoforms of it, e) stimulates β-cat translocation to the nucleus and upregulates mesenchymal markers and WNT target genes f) anchors the WNT signaling protein modulator CXXC4 to the membrane g) induces an “EMT phenotype” in CRC cell lines. To reach these goals, we used both cells that express high levels of NLGN1 physiologically, and cells that were exogenously induced to express NLGN1.

Our results in CRC clinical specimens and cell lines, along with those previously published by Qian et al. [3], consistently show that NLGN1 is expressed in colorectal tumors and may have prognostic implications. Notwithstanding the small percentage of CRC cell lines and clinical samples that display NLGN1 overexpression in our cohorts, we believe that this small subset deserves further characterization. Indeed, NLGN1 may represent a determinant of colorectal metastatic progression in individual patients whereby it could eventually be considered as a novel therapeutic target. In this regard, we note that precision oncology is effective in small subset of patients with colorectal tumors harboring infrequent molecular alterations. This is exemplified by the occurrence of ERRB2 gene amplification, or KRAS G12C mutations, which affect less than 5% and 3% CRCs [9]

Our work is the first to demonstrate that NLGN1 immunohistochemical expression is sustained in high grade tumor budding single cells and in lymphovascular emboli (Fig. 1G, H, I). This is particularly relevant since lymphovascular embolization is a crucial phenomenon, but due to its ephemeral nature, it is difficult to detect on histological sections. Certainly, studies on larger cohorts are needed to validate NLGN1 as a routine marker of tumor budding/EMT and lymphovascular invasion. Regarding the in vitro activity of NLGN1, few reflections must be made. The disruption of endothelial junctions to allow cancer cells to cross the endothelium (TEM) is required for both intravasation and extravasation, even though the two processes are essentially different because the cancer cells approach the endothelium from highly different microenvironments. At the cellular level, our in vitro model of TEM is set up so that cancer cells encounter first the apical side of endothelial cells, mimicking extravasation, in line with the tail vein assay, which shows an increased ability of NLGN1 expressing cells to extravasate and colonize the lungs. However, NLGN1 could also promote intravasation, as it is expressed by single cells in high-grade tumor budding and intravasated emboli (Fig. 1) and it may promote EMT (a necessary step for primary tumor spread and intravasation), as seen by β-cat nuclear translocation and gene expression modulation. Finally, the orthotopic model (Fig. 4) recapitulates all the stages of metastatization and, altogether, we can infer that NLGN1 may be important for both intra and extravasation.

Our next step was to analyze the ability to diffuse and colonize distant organs of CRC cells upon NLGN1 modulation. The tail vein assay has limits as a metastatization assay (tumor cells do not diffuse from a primary tumor but are injected in large numbers into the circulation) but it can be considered an in vivo counterpart of the TEM assay that also evaluates the capacity to colonize an organ. The orthotopic model of CRC presents otherwise the natural progression of a tumor to metastases. Our results show that while the growth of the primary tumors was unaffected, metastatization at particular sites was modulated by NLGN1. The metastatization sites affected by NLGN1 appeared to be different than the liver and lungs, two of the most common sites of CRC metastasis in humans. Given the extreme complexity of the metastatic process, it is impossible to infer on the reasons for the metastatic behavior of our cell models. However, given the vast heterogeneity of CRC, it does not appear surprising that different cell lines may respond differently to NLGN1 modulation in terms of the metastatic site that they colonize. In agreement with this notion, Hugen et al. [38] provide a retrospective review of pathological records of 5817 patients diagnosed with CRC and disclose large differences in metastatic patterns that depend on the histological subtypes and the localization of the primary tumor. Moreover, Riihimäki et al. [39] provide an epidemiologic study of metastatic colon and rectal cancer and reveal the same heterogeneity in the localization of metastatic foci.

Concerning the molecular mechanism of action of NLGN1, we initially evaluated the notion coming from one report from the neurobiology field that APC is required for localizing NLGN1 to neuronal nicotinic synapses [31]. While this function at the synapse may be mechanistically different than that exerted in CRC cells, APC, because of its large size and multi-domain organization, is also a key component of the WNT signaling, which has been the object of high interest in oncology since the first mammalian *WNT* proto-oncogene was discovered [40].

Hence, we first studied the NLGN1/APC membrane localization and NLGN1/APC physical interaction, which supported the result in neurons. Secondly, we studied the nuclear expression of β-cat, the hallmark of active canonical WNT signaling, that is predominant at the invasive front of carcinomas in dissociated, dedifferentiated tumor cells that are at a tumor-host interface and have undergone EMT [41]. As a final proof of NLGN1 involvement in the APC/β-cat pathway we demonstrated that this protein modulates β-cat target genes expression, including EMT markers, while promoting an “EMT phenotype”. Regarding the APC/β-cat pathway it is worth to consider that we used both cells carrying a native form of APC (HuTu 80, SNU-C2A, NCI-H716, HCT116 ) and cells that carry a mutant oncogenic APC (HCT8 cells, carrying missense and non- sense mutations, and HT-29 cells, carrying non-sense mutations, see Supplementary Table S3). In the first group, APC is able to fully participate in the function of the “destruction complex” [22], and in light of our whole set of experiments, NLGN1 appears as an upstream WNT canonical pathway modulator. For the second group, the activity of NLGN1 in the context of mutant oncogenic APC requires some more considerations. First, no noticeable differences in the biological effects of NLGN1 in vitro or in vivo was present between native or mutant APC-carrying cells, APC cortical localization was still induced by NLGN1 in mutant APC bearing cells (our antibody recognizes the mutant forms in HT-29, HCT8, see results section), and in HT-29, NLGN1 co-immunoprecipitated with a band of about 95-100 kDa that is a potential product of the APC mutant E853*. Although lacking mechanistic details, our data point to the idea that NLGN1 and mutant APC could work in synergy, whereby the activities of the APC mutants and NLGN1 would cooperatively increase the aggressivity of cells. Indeed, although mutations in APC are classically thought to lead to a constitutively active WNT pathway that renders cells insensitive to upstream regulation, some experimental data challenge this idea. For example, the WNT antagonists *secreted Frizzled-related proteins (SFRPs)* are able to inhibit Wnt/β-cat signalling in colorectal cancer cell lines carrying APC mutations [42, 43]. Furthermore, previous reports [44, 45] show that in different cell lines, including HT-29, regardless of the extent of the truncation, the APC variants still promote a significant recruitment of β-cat and members of the destruction complex, supporting the hypothesis that the biological effects of NLGN1 in mutant APC carrying cells could still go through the APC/β-cat pathway. It should be considered, for the prosecution of the project, that besides the role in β-cat translocation to the nucleus, NLGN1/APC could affect other cellular functions related to migration/metastatization. Studies have indeed suggested that APC plays roles in cell adhesion and migration and organization of the actin networks through varied mechanisms, possibly independent of the canonical pathway [22, 46]

One final comment on our data can be made concerning the role of neuroligin-3 (NLGN3) in glioma growth. This protein is a member of the NLGN family and is closely related to NLGN1 in terms of structure (72% aa identity and 81% conservation) and function (as they are both present at excitatory synapses). Neuronal activity stimulates NLGN3 extracellular domain shedding into the tumor microenvironment by the protease ADAM10 (A Disintegrin and Metalloprotease 10), thus inducing glioma growth [47]. ADAM10 represents a promising therapeutic target and a clinical trial (#NCT04295759) is ongoing for the inhibition of ADAM10 in glioma with the drug INCB7839 (https://clinicaltrials.gov/). Very interestingly, NLGN1 also undergoes activity-dependent ectodomain shedding by ADAM-10 in the brain [48]. Hence, future research efforts may investigate whether soluble NLGN1 could play a functional role in mediating CRC growth and metastatization.

## Conclusions

We have unveiled NLGN1 as a novel modulator of CRC aggressiveness. We believe that our findings, although do not describe a reciprocal influence between cancer and the nervous system, could be considered part of the rapidly developing “cancer neuroscience” field [49]. Indeed, the expression of neuronal markers in cancer cells facilitates nerve-cancer cross-talk through sharing of common molecular codes, and this perspective is certainly a major possible development of the project.

## Supplementary Information

**Additional file 1: Supplementary Fig. S1.**

**Additional file 2: Supplementary Table S1.**

**Additional file 3: Supplementary Fig. S2.**

**Additional file 4: Supplementary Fig. S3.**

**Additional file 5: Supplementary Fig. S4.**

**Additional file 6: Supplementary Table S2.**

**Additional file 7: Supplementary Table S3.**

**Additional file 8: Supplementary Fig. S5.**

**Additional file 9: Supplementary Fig. S6.**

**Additional file 10: Supplementary Fig. S7.**

**Additional file 11: Supplementary Fig. S8.**

**Additional file 12: Supplementary Fig. S9.**

**Additional file 13: Supplementary Fig. S10.**

**Additional file 14: Supplementary Fig. S11.**

**Additional file 15: Supplementary Material.**

## Data Availability

All data generated or analyzed during this study are included in this published article and its supplementary information files. Further information is available from the corresponding author (marco.arese@unito.it) upon request.

## Abbreviations

APC: Adenomatous Polyposis Coli
β-cat: Catenin Beta 1
*c-MYC*: Proto-Oncogene C-Myc
CDK1a: Cyclin Dependent Kinase 1α
CK1: Casein Kinase 1 α1
*DVL*: Dishevelled
*EGF*: Epidermal Growth Factor
*FZD*: Frizzled receptor
GSK3Beta: Glycogen Synthase Kinase 3 Beta
*L1CAM*: L1 Cell Adhesion Molecule
*LAMC2*: Laminin Subunit Gamma 2
*LRP*: LDL Receptor Related Protein
*MMP*: matrix metalloproteinase
*TGFβ*: Transforming Growth Factor Beta
*Twist*: Twist Family BHLH Transcription Factor
*uPA*: Plasminogen Activator, Urokinase
*uPAR*: Plasminogen Activator, Urokinase Receptor
