# Supplementary figures and images for: The neuronal protein Neuroligin 1 promotes colorectal cancer progression by modulating the APC/β-catenin pathway
Source: J Exp Clin Cancer Res. 2022 Sep 2;41:266. doi: 10.1186/s13046-022-02465-4 (PMC9438340; doi:10.1186/s13046-022-02465-4)

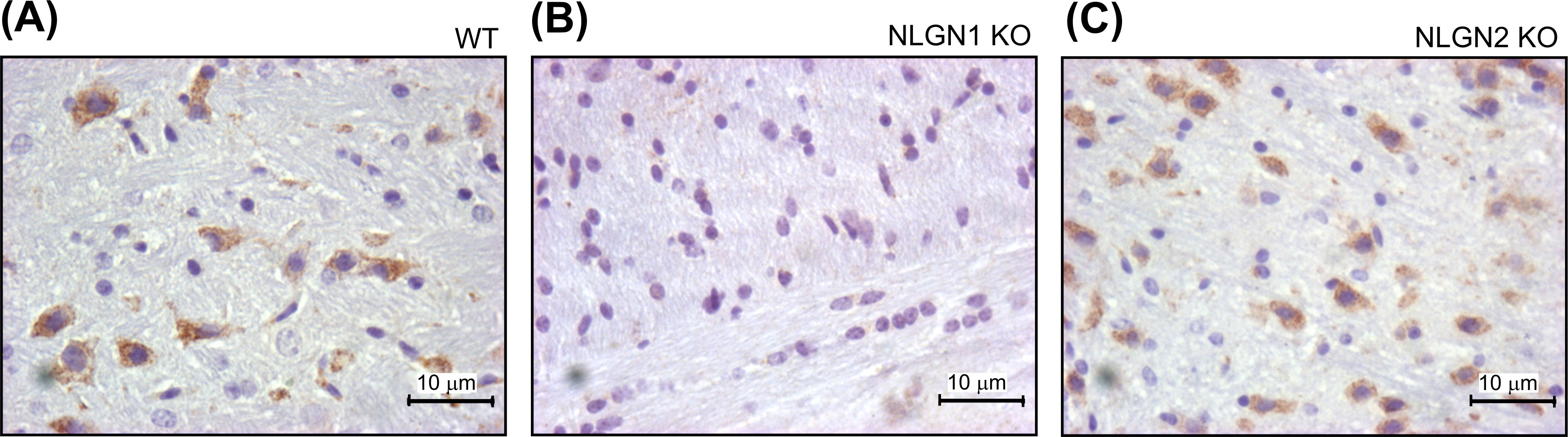

Supplement: Supplementary file 1 — Additional file 1: Supplementary Fig. S1. [file 13046_2022_2465_MOESM1_ESM.jpg]

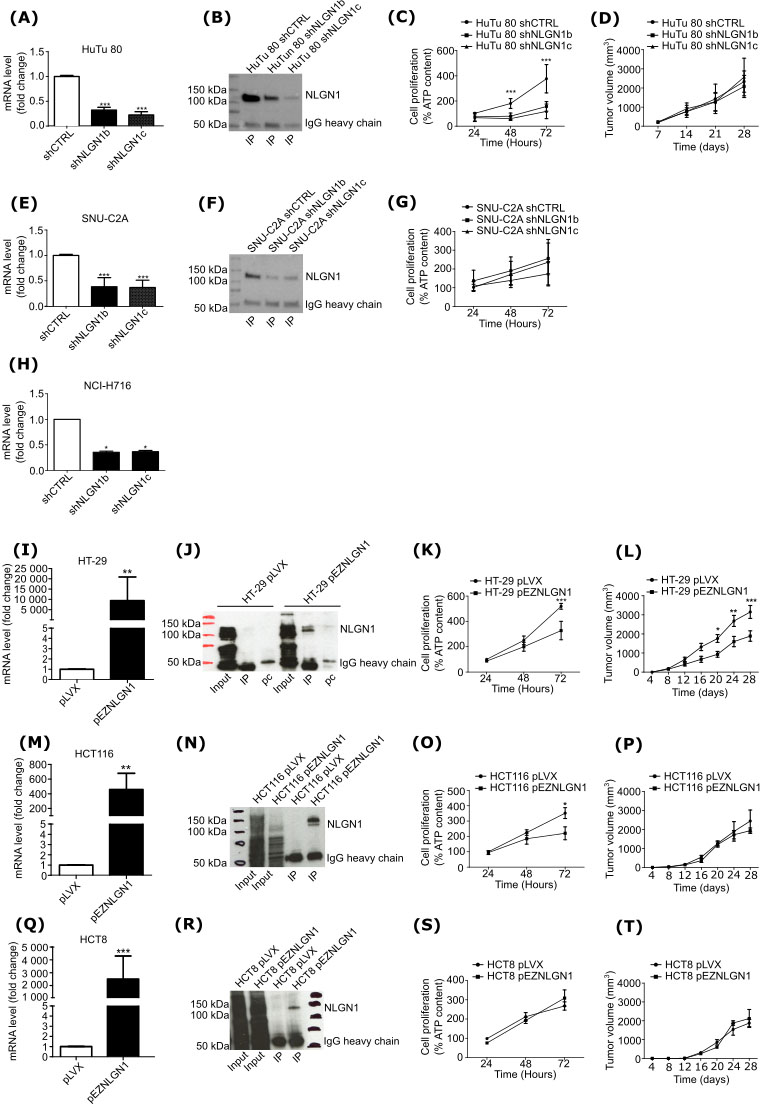

Supplement: Supplementary file 3 — Additional file 3: Supplementary Fig. S2. [file 13046_2022_2465_MOESM3_ESM.jpg]

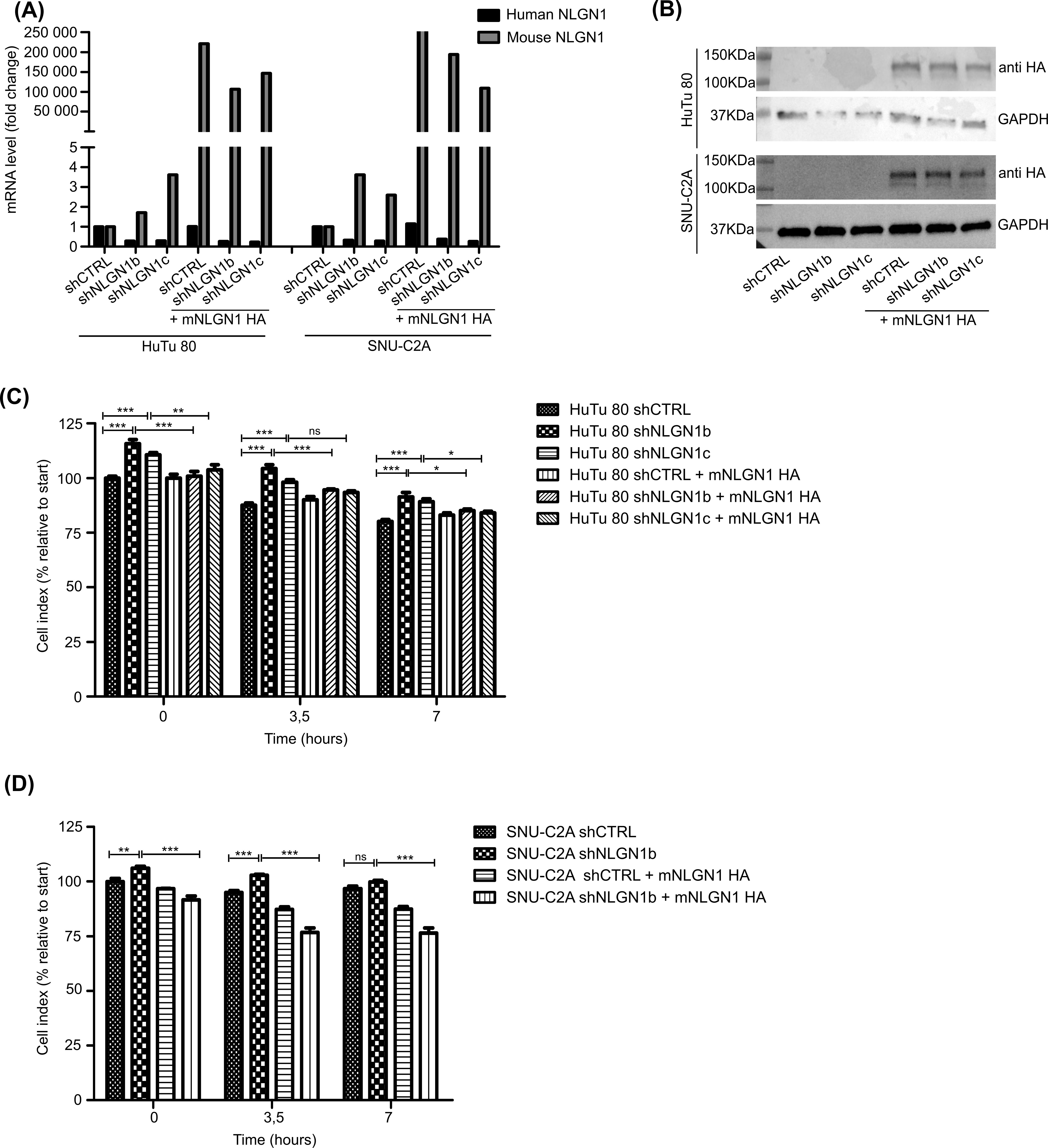

Supplement: Supplementary file 4 — Additional file 4: Supplementary Fig. S3. [file 13046_2022_2465_MOESM4_ESM.jpg]

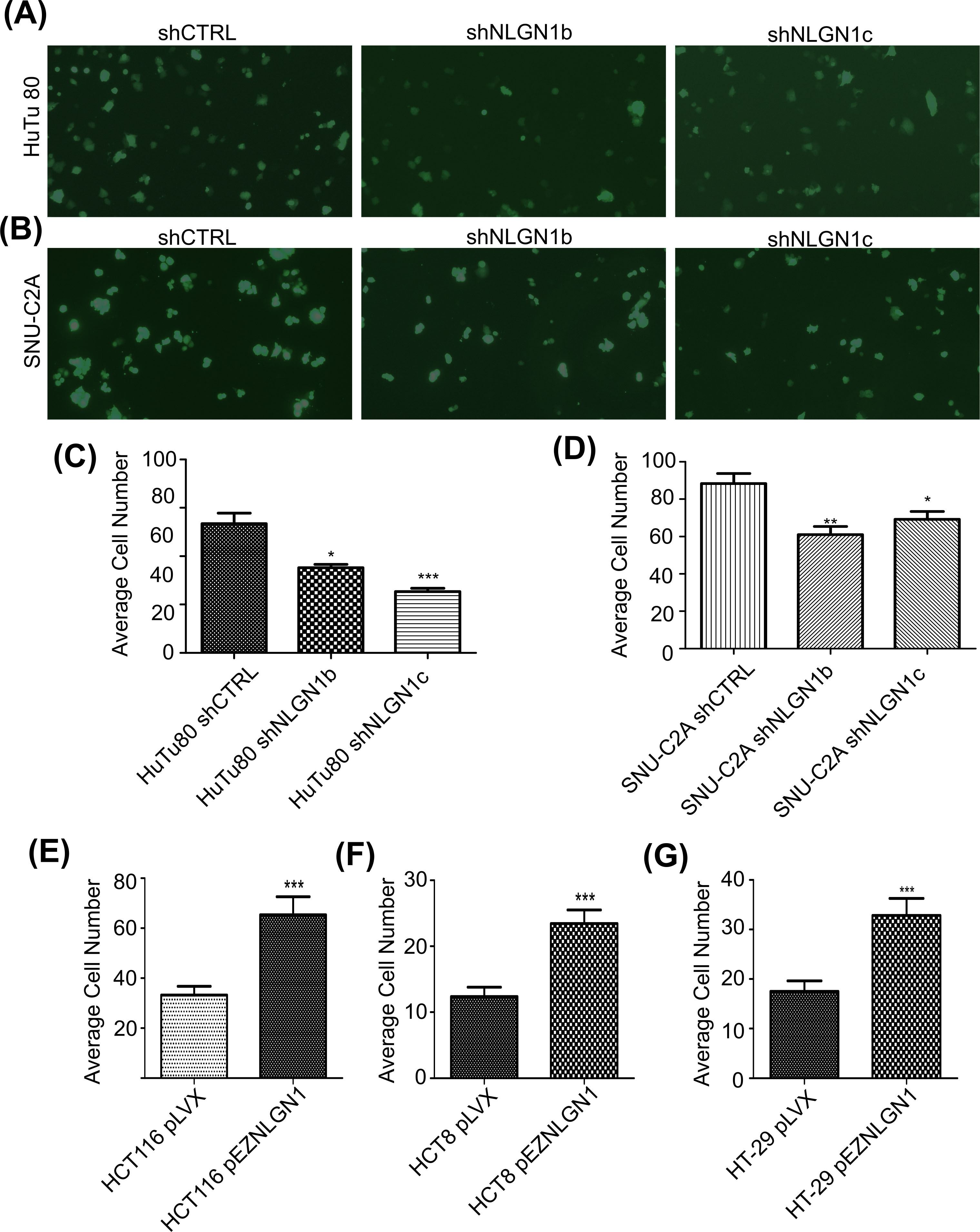

Supplement: Supplementary file 5 — Additional file 5: Supplementary Fig. S4. [file 13046_2022_2465_MOESM5_ESM.jpg]

**Supplementary Table S2:** WNT pathway genes correlated with NLGN1


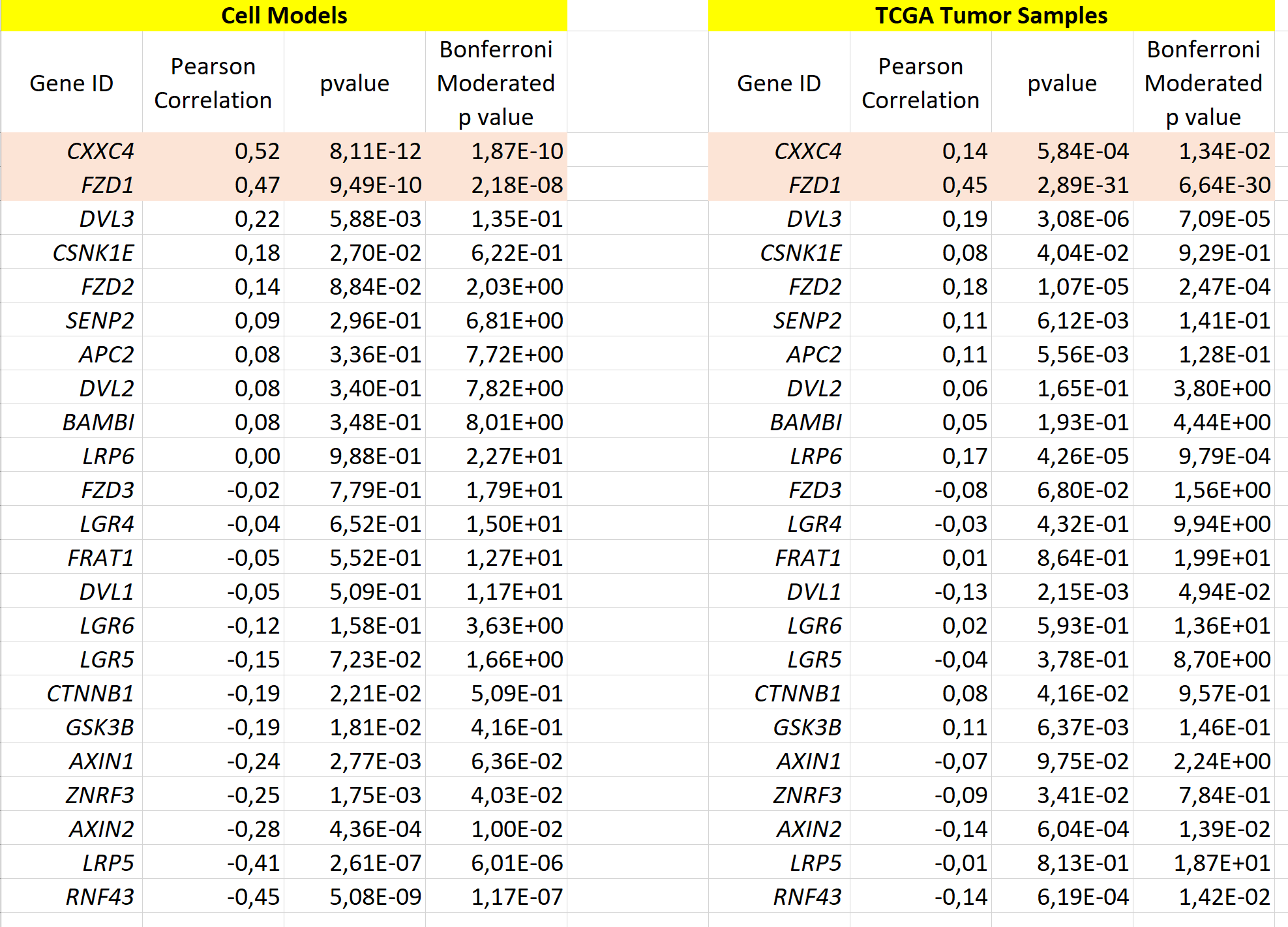

Supplement: Supplementary file 6 — Additional file 6: Supplementary Table S2. [file 13046_2022_2465_MOESM6_ESM.docx]

Supplementary table S3: APC mutation in CRC cell lines


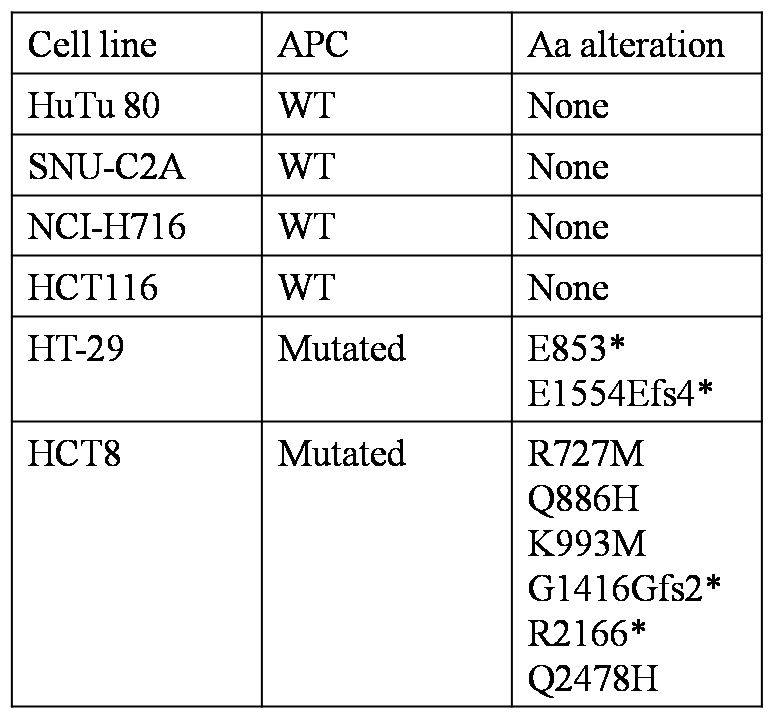

Supplement: Supplementary file 7 — Additional file 7: Supplementary Table S3. [file 13046_2022_2465_MOESM7_ESM.docx]

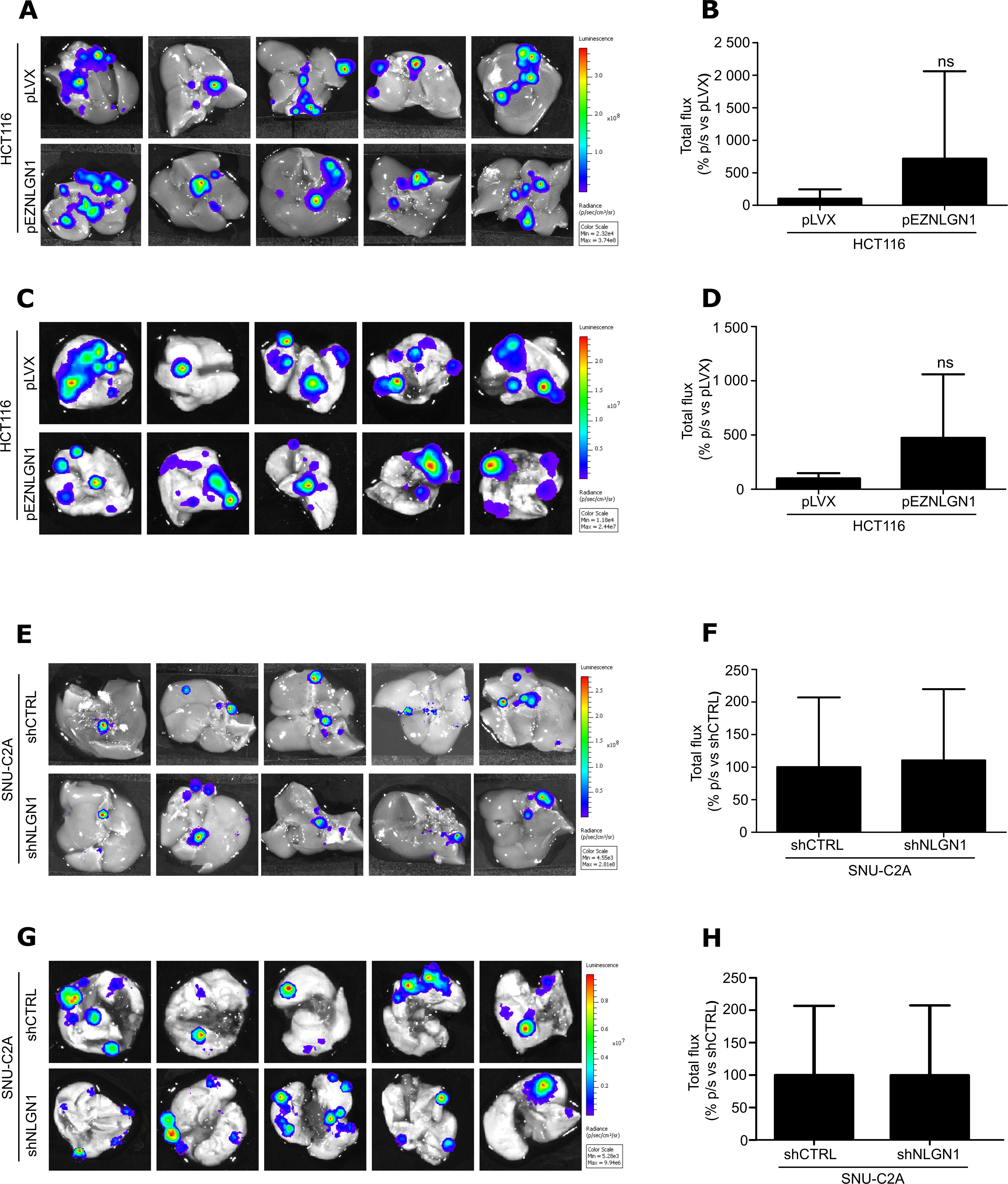

Supplement: Supplementary file 8 — Additional file 8: Supplementary Fig. S5. [file 13046_2022_2465_MOESM8_ESM.jpg]

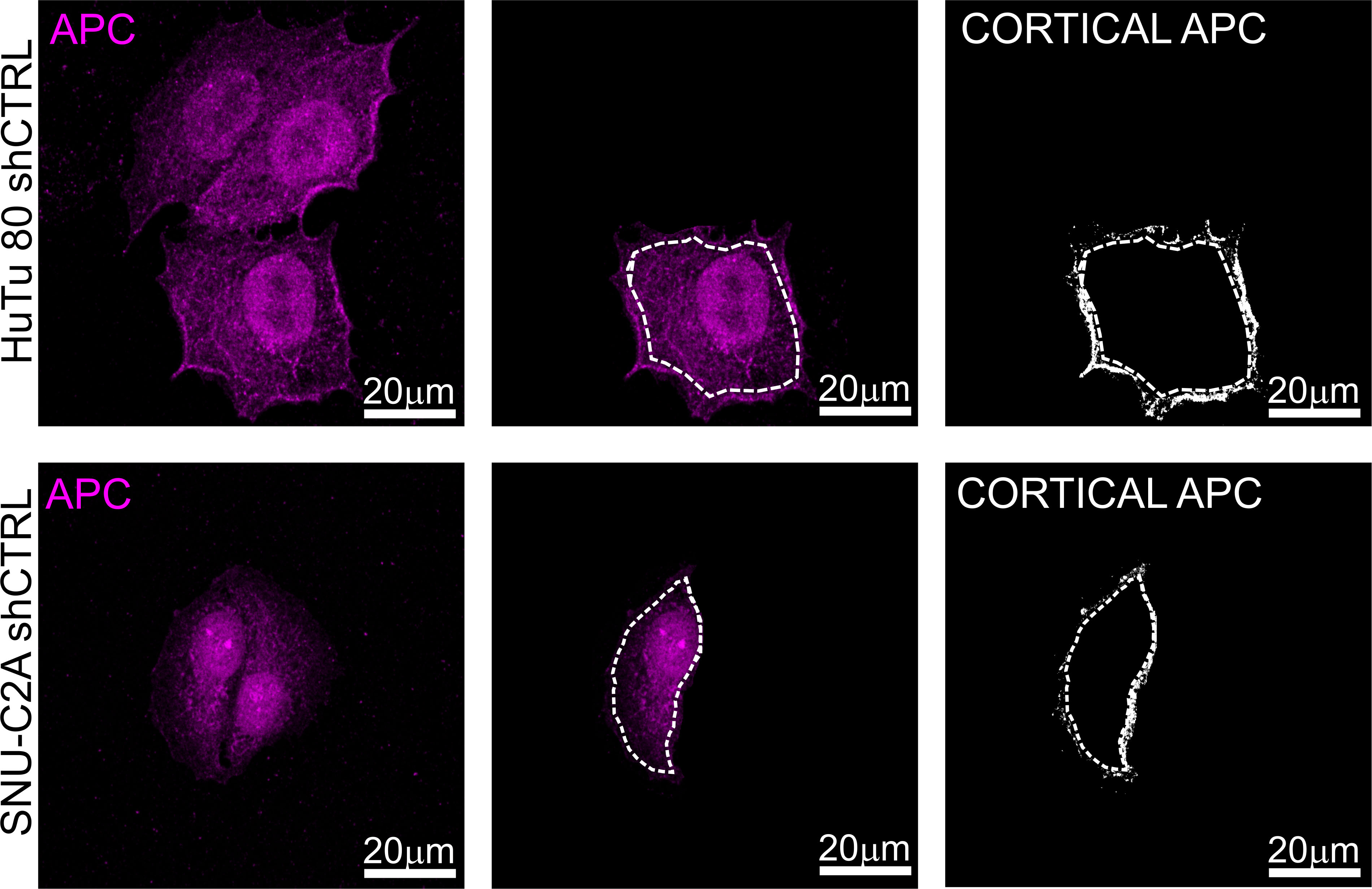

Supplement: Supplementary file 9 — Additional file 9: Supplementary Fig. S6. [file 13046_2022_2465_MOESM9_ESM.jpg]

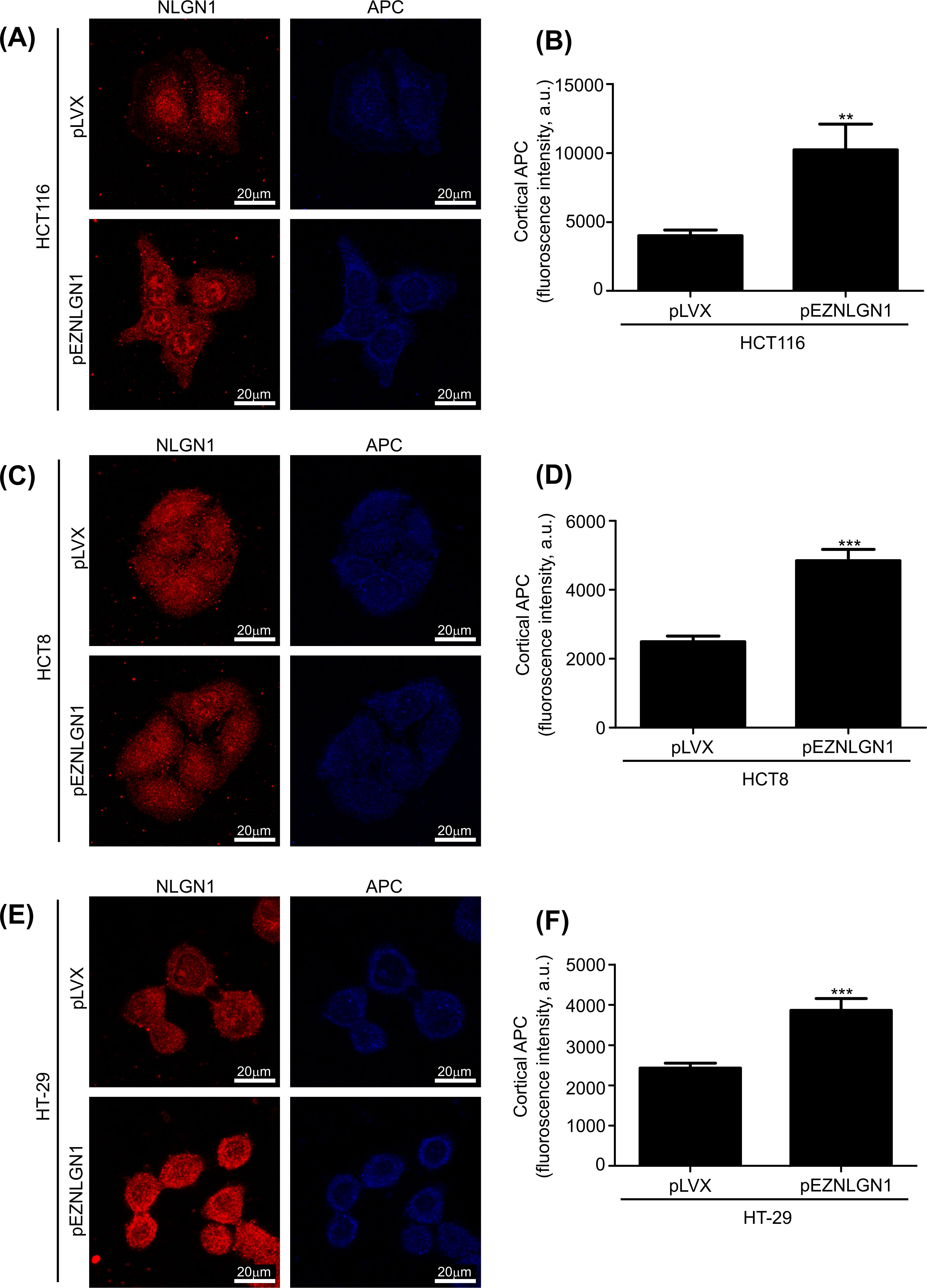

Supplement: Supplementary file 10 — Additional file 10: Supplementary Fig. S7. [file 13046_2022_2465_MOESM10_ESM.jpg]

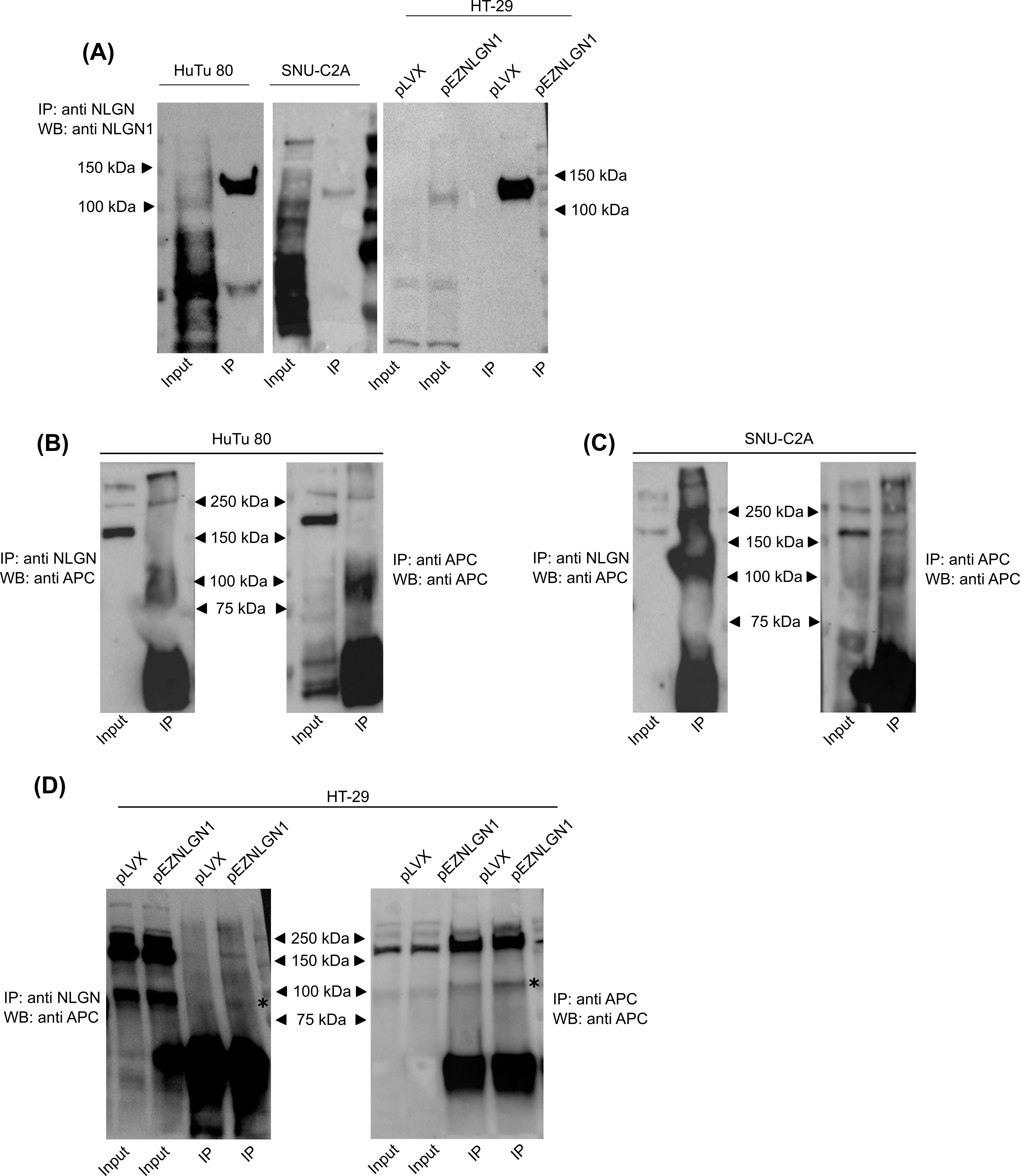

Supplement: Supplementary file 11 — Additional file 11: Supplementary Fig. S8. [file 13046_2022_2465_MOESM11_ESM.jpg]

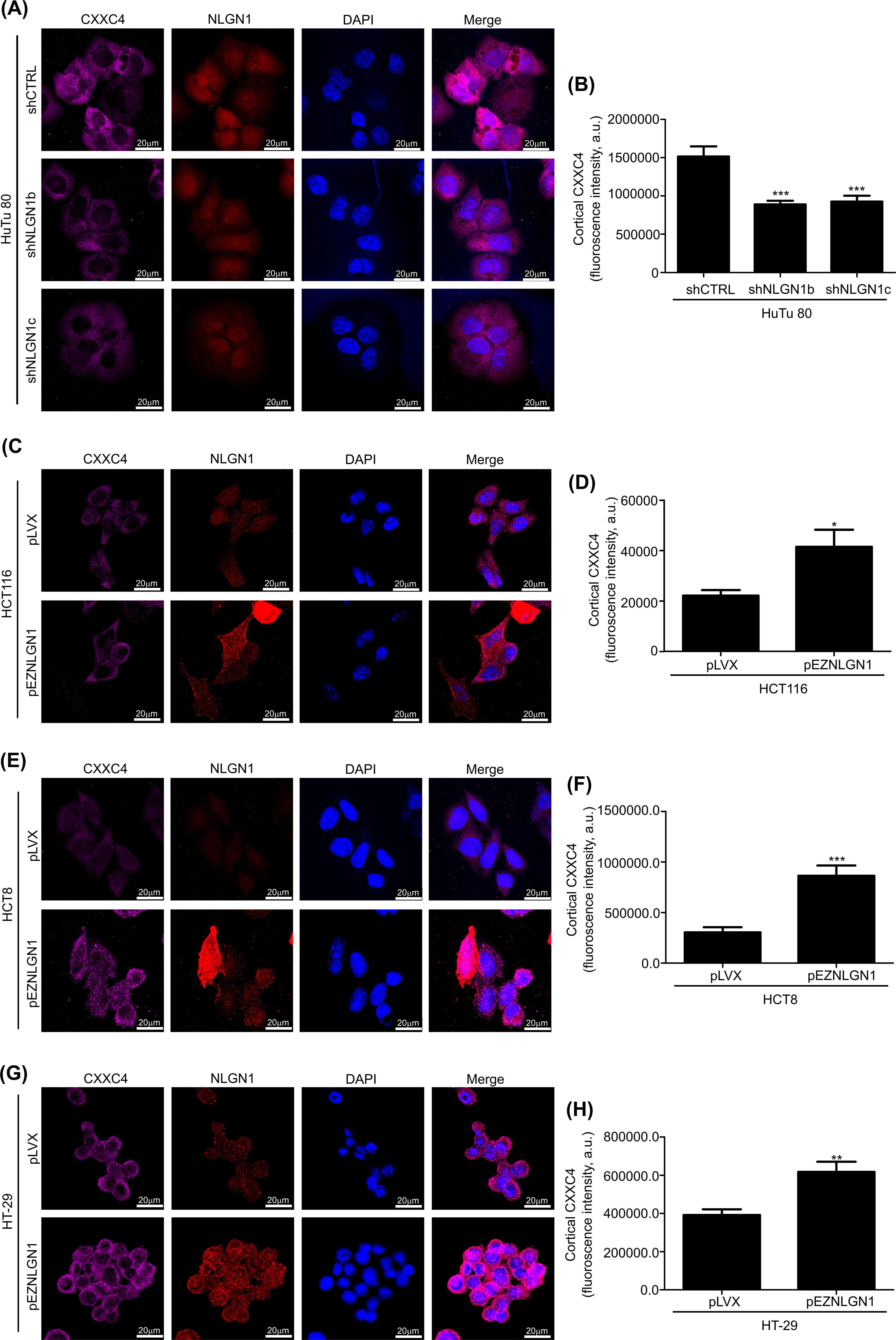

Supplement: Supplementary file 12 — Additional file 12: Supplementary Fig. S9. [file 13046_2022_2465_MOESM12_ESM.jpg]

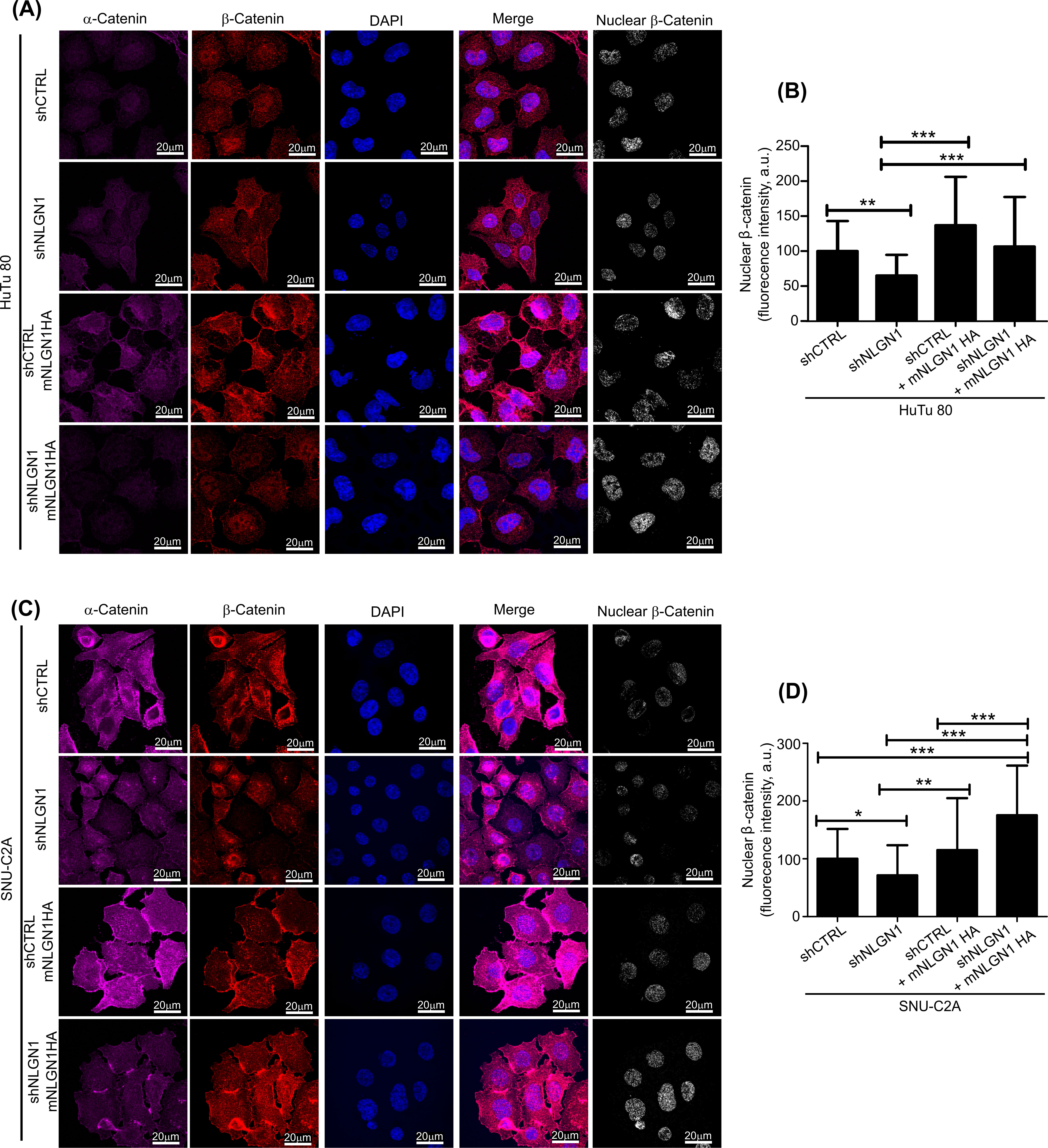

Supplement: Supplementary file 13 — Additional file 13: Supplementary Fig. S10. [file 13046_2022_2465_MOESM13_ESM.jpg]

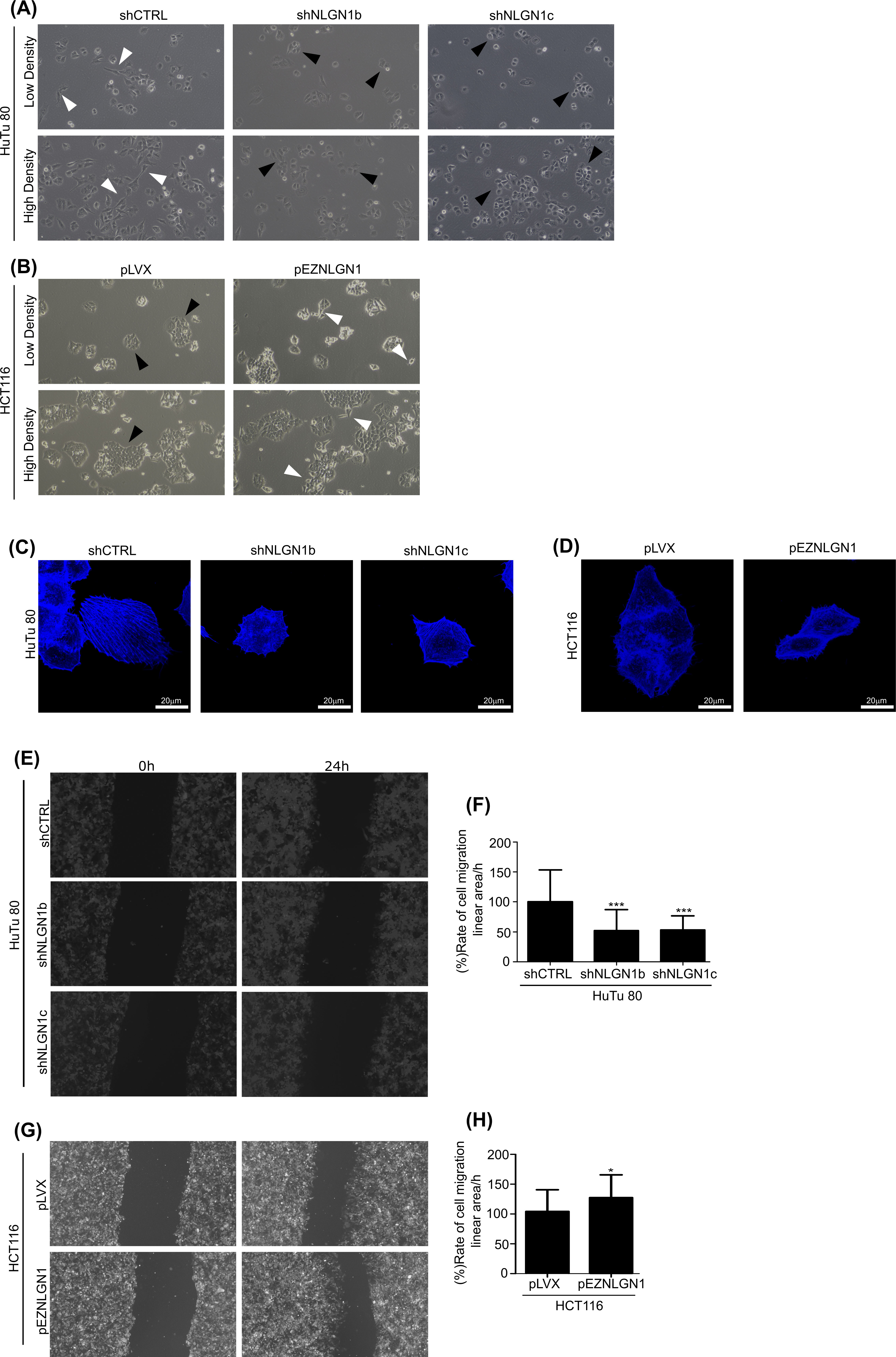

Supplement: Supplementary file 14 — Additional file 14: Supplementary Fig. S11. [file 13046_2022_2465_MOESM14_ESM.jpg]
