# Supplementary material for: The neuronal protein Neuroligin 1 promotes colorectal cancer progression by modulating the APC/β-catenin pathway
Source: J Exp Clin Cancer Res. 2022 Sep 2;41:266. doi: 10.1186/s13046-022-02465-4 (PMC9438340; doi:10.1186/s13046-022-02465-4)

**Supplementary Table S1: Cell ID.** Cell-line STR profiles were cross-compared and matched with the available STR from ATCC online database. This corresponds to a percentage of identity of: 100% for HT-29, HCT116 and NCI-H716; 94% for HCT8 and SNU-C2A; 82% for HuTu 80.


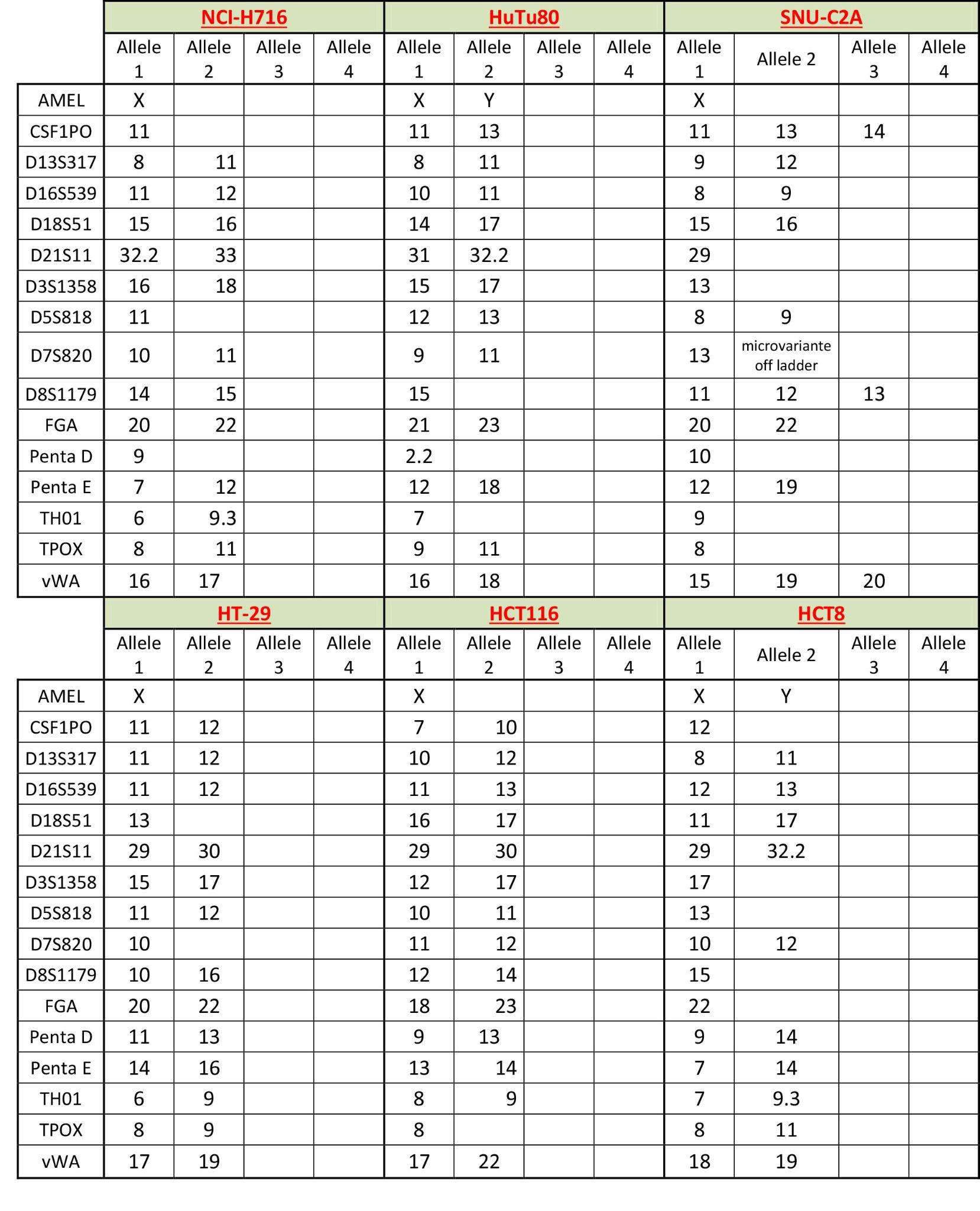

Supplement: Supplementary file 2 — Additional file 2: Supplementary Table S1. [file 13046_2022_2465_MOESM2_ESM.docx]
