# Supplementary material for: The neuronal protein Neuroligin 1 promotes colorectal cancer progression by modulating the APC/β-catenin pathway
Source: J Exp Clin Cancer Res. 2022 Sep 2;41:266. doi: 10.1186/s13046-022-02465-4 (PMC9438340; doi:10.1186/s13046-022-02465-4)
Supplement: Supplementary file 15 — Additional file 15: Supplementary Material. [file 13046_2022_2465_MOESM15_ESM.docx]

**Supplementary information**

**Supplementary Materials and Methods**

***Antibodies***

The antibodies used in this study were: the rabbit anti-NLGN antibody L067 (rabbit immunization with PHPHPHSHSTTRV peptide) from New England Peptide (Boston, MA, USA); anti-NLGN1 Mouse monoclonal purified IgG (clone 4C12) from Synaptic System (Gottinga, Germany); Anti-NLGN1 Mouse monoclonal Neuromab (clone N97A/31) from ORIGENE (Rockville, MD, USA); Alpha catenin Mouse monoclonal Antibody (alpha-CAT-7A4) from Thermofisher Scientific (Waltham, MA, USA); Rabbit Polyclonal Anti-beta Catenin antibody (ab6302), Recombinant Rabbit Monoclonal Anti-APC antibody [EP701Y] (ab40778), Rabbit polyclonal Anti- CXXC4 (Ab105400) and Mouse monoclonal GAPDH (6C5)(ab8245) from Abcam (Cambridge, UK); Rabbit Monoclonal β-actin (13E5) and Rabbit Monoclonal anti-HA (3725S) from Cell Signaling ([Danvers,](https://www.google.com/search?sxsrf=AOaemvJQmW_Q5ds_Bwf1txptqWMRp8Rg7w:1642417489816&q=Danvers&stick=H4sIAAAAAAAAAOPgE-LSz9U3MCu3KKlIUeIEsQ1zKsrztLSyk63084vSE_MyqxJLMvPzUDhWGamJKYWliUUlqUXFi1jZXRLzyoCsHayMAAj2gl1SAAAA&sa=X&ved=2ahUKEwirgLb40bj1AhWugVwKHZH5ACIQmxMoAXoECCkQAw)  MA, USA); mouse monoclonal Anti-APC (F-3) antibody (sc-9998) from Santa Cruz Biotechnology (Dallas, TX, USA)..

***Immunohistochemistry on brains of WT, NLGN1 ^-/-^ and NLGN2 ^-/-^null mice.***

NLGN1 antibody (ORIGENE) was validated by immunohistochemistry (IHC) on brains of WT, NLGN1*^-/-^* and NLGN2*^-/-^* null mice. Brains of sacrificed animals were formalin fixed, paraffin embedded and 5μm tissue sections were obtained using RM2235 *Leica* microtome. IHC was performed following ORIGENE protocol with few modifications. Slides were heated in an oven at 65°C for 1 hour, de-paraffinized and rehydrated. Heat-induced antigen retrieval was performed in Citrate buffer 1x (Thermofisher scientific) for 30 minutes in a 95°C water bath. Endogenous peroxidase were inactivated by covering tissue with 3% hydrogen peroxide (SIGMA) for 10 min. Then slides were washed three times with TBST and saturated with blocking solution containing 10% Normal goat serum (Vector labs, Burlingame, CA, USA) for 1h at room temperature.The tissues were then incubated overnight in 4°C humidified chamber with anti-NLGN1 antibody (1:200 Anti-NLGN1 Mouse monoclonal Neuromab (clone N97A/31) from ORIGENE) diluted in blocking solution containing 10% Normal goat serum, washed 3 times in TBS-T, and incubated for 1h with anti-mouse HRP-conjugate antibody in blocking solution containing 10% Normal goat serum. The immunochemical reaction was visualized with the DAB kit (Dako, Santa Clara, CA, USA). The tissues were counterstained with Mayer’s hematoxylin (Bio-Optica) and mounted on glass. Optical images were taken with the Leica DM2000 microscope.

***RNA extraction, retrotranscription and qRT-PCR***

The total RNA was extracted using Maxwell RSC simplyRNA Tissue Kit (Promega, Madison, WI, USA) according to the manufacturer’s instructions. The mRNAs obtained were quantified with the DeNovix DS-11+ Spectrophotometer. The quality of RNA was checked by the Agilent 2100 bioanalyzer (Agilent Technologies Waldbronn, Germany). First-strand cDNA was generated from 1μg of total RNA using the High Capacity cDNA Reverse Transcription Kit (Applied Biosystem, [Waltham,](https://www.google.com/search?sxsrf=AOaemvJRKZ0xeybGHi6JIZ8IhXIxchOm0A:1642419033566&q=Waltham&stick=H4sIAAAAAAAAAOPgE-LSz9U3MKoyzMkuUuIAsTOqjE21tLKTrfTzi9IT8zKrEksy8_NQOFYZqYkphaWJRSWpRcWLWNnDE3NKMhJzd7AyAgB_zvC8UQAAAA&sa=X&ved=2ahUKEwi0_sTY17j1AhWk7rsIHUnkBkUQmxMoAXoECBQQAw&biw=1517&bih=694&dpr=0.9) MA, USA). The expression of human NLGN1 (Hs00208784_m1), MYC (Hs00153408_m1), Cyclin D1 (Hs01050839_m1),Twist (Hs00361186_m1), L1CAM ([Hs01109748_m1](https://www.thermofisher.com/taqman-gene-expression/product/Hs01109748_m1?CID=&ICID=&subtype=)), LAMC2 ([Hs01043717_m1](https://www.thermofisher.com/taqman-gene-expression/product/Hs01043717_m1?CID=&ICID=&subtype=))and *M. musculus* NLGN1 (Mm02344305_m1) was analyzed by quantitative real-time reverse transcription-PCR (qRTPCR) using TaqMan Gene Expression Assay (Applied Biosystem, Waltham, MA, USA). Instead the expression of N-cadherin (forward 5’-CTCCAGGGGACCTTTTCCT-3’;  reverse 5’-CCGAGATGGGGTTGATAATG-3’)  and MMP2 (forward 5’-AGACCGCCATGTCCACTGTT-3’; reverse 5’-TGGTCGCACACCACATCTTT-3’) was analyzed by quantitative real-time reverse transcription-PCR (qRTPCR) using PowerUp SYBR Green Master (Applied Biosystem, Waltham, MA, USA). The mRNA levels, analyzed in triplicate, were normalized against human (Hs00427620_m1) TATA-binding box protein (TBP) and human Actin beta (forward 5’-ACAGAGCCTCGCCTTTG-3'; reverse 5’-CCTTGCACATGCCGGAG-3') as control genes. The fold increase or decrease was determined relatively to the control through the use of the formula 2^-ΔΔCT^.

### ***Immunoprecipitation, Coimmunoprecipitation and immunoblotting analysis***

### Subconfluent CRC cell lines were homogenized in cold EB buffer (10 mm Tris-HCl, pH 7.5, 150 mm NaCl, 5 mm EDTA, pH 8; 10% glycerol, 1% Triton X-100, protease and phosphatase inhibitors, 50 μg/ml of pepstatin, 50 μg/ml of leupeptin, 10 μg/ml of aprotinin, 1 mm PMSF, 100 μm ZnCl2, 1 mm sodium orthovanadate, and 10 mm NaF). After centrifugation (20 min, 4 °C at 10,000 × *g*), the supernatants were quantified with BCA Protein Assay Reagent Kit (Pierce Chemical Co., Dallas, TX, USA). Proteins for each sample were pre-cleared 1h incubation with A-Sepharose protein (Amersham Biosciences, Amersham, UK). Each sample was then incubated overnight at 4 °C with rabbit polyclonal anti-NLGN antibody (L067) used at a final concentration of 2.5 μg/mg of protein. The immune complexes were recovered on protein A-Sepharose for 1 h and 30 min, pelleted, and washed four times with lysis buffer. The proteins were separated by 10% SDS-PAGE electrophoresis gel, transferred to polyvinylidenedifluoride (PVDF), and immunodecorated with  a mouse monoclonal anti-NLGN1 antibody (4C12 1:1000).

The coimmunoprecipitation was performed as described above using both rabbit polyclonal anti-NLGN antibody (L067) and recombinant anti-APC antibody [EP701Y] (Ab40778 Abcam) used at a final concentration of2μg/mg of protein.

 For the Western blotting analysis PVDF membranes were immunodecorated with mouse monoclonal anti-NLGN1 antibody (4C12, 1:1000) and recombinant anti-APC antibody [EP701Y] antibody (1:1000). Secondary antibodies were HRP-conjugated anti-mouseand anti.rabbit(Jackson ImmunoResearch). Antibodies were detected with ECL reagent (Amersham/GE Healthcare, Amersham, UK).

***CRC cell cultures, overexpression, silencing and DNA constructs***

All CRC cell lines used in this paper are part of Candiolo Institute CRC Bank. HT-29 (ATCC), HCT116 (ATCC), HCT8 (ATCC), NCI-H716 (ATCC) and SNU-C2A (Korean cell line bank) were grown in 10% FBS RPMI; HuTu 80(ATCC) were grown in 10%FBS MEM. The identity of each cell line was checked by Cell ID System and by Gene Print 10 System (Promega), through STR at 10 different loci (D5S818, D13S317, D7S820, D16S539, D21S11, vWA, TH01, TPOX, CSF1PO and amelogenin). Resulting cell-line STR profiles were cross-compared and matched with the available STR from ATCC online database. All cell lines were tested for mycoplasma regularly with Venor GM Kit (Minerva Biolabs, Berlin, Germany). Stable NLGN1 overexpressing HT-29, HCT116, HCT8 and relative control were generated by infection with lentiviral vectors pEZ-Lv105 NLGN1(pEZ-Lv105 expression vector, GeneCopoeia) and PLVX. NLGN1 silenced SNU-C2A, HUTU80 and NCI-H716 cell populations and relative control were generated by infection with two lentiviral vectors targeting NLGN1 from Origene: cat # TL3111634B (shNLGN1b) and cat # TL3111634C (shNLGN1c) or control cat # TR30021 (shCTRL) (pGFP-C-shLenti shRNA-29mer expression vector-ORIGENE, Rockville, MD, USA). For all the experiments in vitro and in vivo shNLGN1b (shNLGN1) was used preferentially. Cells were seeded at concentrations of 5 × 10^5^ cells/ml in a 10-cm/diameter tissue culture dish, transduced for 36 h with lentiviral particles in the presence of 8μg/ml of Polybrene (Sigma-Aldrich, St. Louis, MO, USA) and selected for 48 h with 2 mg/ml of puromycin. The efficiency of NLGN1 expression modulation for all systems was determined by qRTPCR analysis and immunoprecipitation/WB and shown in supplementary Figure S2.

***Cell proliferation***

Cells were seeded at the concentration of 3000 cells/well in 96-well plates in the appropriate culture medium. Cell proliferation was measured at 0, 24, 48 and 72 hours by CellTiter-Glo Luminescent Assay kit (G7572, Promega).

***In vivo xenografts tumor growth***

6 week-old NOD/SCID mice were injected subcutaneously with 10 × 10^6^ HuTu 80, HT-29, HCT116 or HCT8 cells in the lower-right flank. Tumour growth was monitored once a week by caliper measurement. Tumor size was expressed in mm^3^ using the standard formula: length × width^2^ × 0.52.

### ***Immunofluorescence and quantification***

### *Alpha/beta catenin stainings*: cells, plated on glass coverslips, were fixed for 10 minutes with 4% paraformaldehyde solution (Santa Cruz Biotechnology, Dallas, TX, USA), permeabilized in PBS 0.1% Triton-X100 for 15 minutes at room temperature, blocked in PBS 2% BSA, 10% donkey serum (Sigma-Aldrich, St. Louis, MO, USA), 0.3M glycine and 0.1% tween for 2 hour at room temperature and then incubated in the same blocking buffer with anti-alpha catenin (1:250) and anti-β-cat (1:200) antibodies overnight at 4°C.*NLGN1/APC/CXXC4stainings*: HuTu 80 and SNU-C2A cells, plated on glass coverslips, were fixed for 6 minutes with 4% paraformaldehyde solution (Thermo Scientific Waltham, MA, USA), permeabilized in PBS 0.5% Saponin for 6 minutes at room temperature, blocked in PBS, 0.2% Saponin, 5% Fetal bovine serum, 10% donkey serum,1% BSA, 0.3 M glycine and 0.1% tween and incubated with the anti-APC antibody from AbCam (1:50) or anti-CXXC4 from AbCam (20µg/ml) in the same blocking buffer overnight at 4°C. The day after cells were incubated with an anti-NLGN1 antibody from R&D (1:50) for 1h at room temperature. HCT116, HCT8 and HT-29 cells, plated on glass coverslips,were fixed in MeOH for 10 minutes at -20°C,blocked in PBS 10% Donkey Serum, 1% BSA, 0.3% Triton-X, 0.3M Glycinand incubated with the anti-APC antibody from Santa Cruz (1:50) or anti-CXXC4 from AbCam (20µg/ml)and with the polyclonal anti-NLGN1 from Origene (1:50) in the same blocking buffer overnight at 4°C. Primary antibodies were revealed by appropriate Alexa Fluor 647- and 555-conjugated secondary antibodies (1:400) for 1h at room temperature.Immunofluorescence images were taken with the Leica SPE microscope and the LasAF software (Leica Microsystem, Wetzlar, Germany). Nuclear β-cat was measured as difference of total β-cat and α-cat with ImageJ (*OriolArqués, Irene Chicote, Stephan Tenbaum, Isabel Puig and Héctor G. Palmer Quantitative Procedure to Analyze Nuclear β-Catenin Using Immunofluorescence Tissue Staining Technical Report in Protocol Exchange June 2014 ISSN 2043-0116 DOI: 10.1038/protex.2014.018*). APC levels were analyzedat the cell cortical level, manually identified through the line tool, using ImageJ.

***Manual in vitro transendhothelial migration quantification***

1.4 x 10^5^ HUVEC cells were seeded on matrigel onto each well of a 24 MW and allowed toreach confluency. Then HUVEC media was replaced by 500 μl of GFP- positive tumor cells (4 x 10^4^ cells) resuspended in the appropriated culture media.Tumor cells adhesion was monitored for 5 hours and photographed using a ZEISS Axio Vert.A1 microscope with a True Chrome HDII camera. Four representative images were taken for each well and every experimental point was in quadruplicate. Fluorescent cells were then manually counted using the ImageJ Software.

### ***Nuclear protein extraction and immunoblotting analysis***

### The nuclear extraction was prepared using an NE-PER Nuclear Cytoplasmic Extraction Reagent kit (Thermo Scientific Waltham, MA, USA) according to the manufacturer's instruction. Briefly, cells were washed twice with cold PBS and centrifuged at 500 *g* for 3 min. The cell pellet was suspended in 200 μl of cytoplasmic extraction reagent I by vortexing. The suspension was incubated on ice for 10 min followed by the addition of 11 μl of a second cytoplasmic extraction reagent II, vortexed for 5 s, incubated on ice for 1 min and centrifuged for 5 min at 16 000 *g*. The supernatant fraction (cytoplasmic extract) was transferred to a pre-chilled tube. The insoluble pellet fraction, which contains crude nuclei, was resuspended in 100 μl of nuclear extraction reagent by vortexing during 15 s and incubated on ice for 10 min, then centrifuged for 10 min at 16 000 *g*. The proteins in the resulting supernatant, constituting the nuclear extract, were separated by 4-12% SDS-PAGE electrophoresis gel, transferred to a  nitrocellulose  membrane (Biorad, Hercules, CA, USA) and then immunodecorated with specific antibodies against Anti-beta Catenin, beta-actin and GAPDH. Secondary antibodies were HRP-conjugated goat anti-rabbit or anti-mouse (Jackson ImmunoResearch, Cambridgeshire, UK). Antibodies were detected with ECL reagent (Amersham/GE Healthcare,Amersham, UK).

### ***cDNA Transient Transfection***

### Stable NLGN1 silenced SNU-C2A and HuTu 80 cells (shNLGN1b; shNLGN1c) and relative control (shCTRL) were transient trasfected with the pCAG vector containing the HA-tagged Musmusculus NLGN1 construct (mNLGN1HA). Briefly CRC cells (2x10^5^ cells/well) were seeded in 6-well plates and transfected in Opti-MEM I ( Gibco) with 5 μg of DNA by using the Lipofectamine™ Transfection Reagent (Invitrogen™). After 3 h at 37 °C the Opti-MEM I was replaced with CRC cells medium. 48h later RNA samples and protein lysates were collected and checked for mNLGN1HA expression both by qRTPCR with human and mouse NLGN1 probe and western blotting with the rabbit anti-HA antibody. In parallel cells for α/β catenin immunofluorescence were prepared.

***Cell Morphology analysis***

5 x 10^4^  (low density) or 1.5 x 10^5^  (high density) cells/well were plated in a 12-well plate and cultured in 10% FBS culture media at 37°C . Images were taken at 24 and 48h using a ZEISS Axio Vert.A1 microscope with a True Chrome HDII camera.

###### **Scratch assay**

A scratch assay was performed to evaluate the migration of cells. 5 x 10^5^ cells/well were seeded in a 96-well plate. When cells reached the 100% of confluence  a sterile 200 µl pipette tip was used to create a straight scratch on the surface of the cell layer and the cells were  carefully rinsed with culture medium to remove free-floating cells and debris and  cultured for 24h in a 2% FBS culture medium.   Scratch zones, representative for each cell line,were photographed at 0h and 24h by a ZEISS Axio Vert.A1 microscope with a True Chrome HDII camera (20x).  The scratch widths modifications were calculated as rate of cell migration linear area/hby subtracting the linear measure of 24h scratch to the linear measure of 0h over 24.

**Supplementary figure legends**

**Supplementary Figure S1:** Anti-NLGN1 antibody validation. IHC performed using the anti-NLGN1 Mouse monoclonal Neuromab (clone N97A/31) from ORIGENE on slices derived from WT (A), NLGN1 knockout (B) and NLGN2 knockout mice. Scale bar: 10 μm.

**Supplementary Figure S2:** NLGN1 modulation. Expression level of NLGN1, evaluated through qRT-PCR **(A, E, H, I, M and Q)**, immunoprecipitation assay **(B, F, J, N and R),** cell proliferation in vitro **(C, G, K, O and S)** and tumor growth in vivo **(D, L, P and T)**, in HuTu 80 **(A-D)**, SNU-C2A **(E-G)** and NCI-H716 **(H)** control (shCTRL) cells or in with NLGN1 is downregulated with two specific short hairpin RNA sequence targeting NLGN1 (shNLGN1b and c) and HT-29 **(I-L)**, HCT116 **(M-P)** and HCT8 **(Q-T)**transductedwith the lentiviral vector PLVX empty (pLVX) or with a lentiviral vector containing the human cDNA sequence of NLGN1 (pEZNLGN1). qRT-PCRwas performed using TaqMan Gene Expression Assay. Fold-change was calculated with respect to control cells and values are expressed as mean ± SE (*n* = 3 independent experiments). One-way ANOVA with Bonferroni test **(A, E and H)** or Unpaired Student's t test, two tailed **(I, M and Q)**: * p < 0.05 **, p < 0.01, *** p < 0.001. Immunoprecipitationwas performed using an anti-NLGN (L067) antibody and immunoblotting was conducted with a monoclonal antibody (4C12) able to recognize NLGN1. The 120-kDa band detectable in the IP lanes corresponds to NLGN1. The images shown are representative of 1 out of 3 reproducible experiments. Cell proliferation was assessed by the Cell Titer Glo assay. Graphs in **C**, **G**, **K**, **O**, **S**  show the luminescence as arbitrary units and values are expressed as mean ± SD (n=3 independent experiments performed in triplicate). One-way ANOVA with Bonferroni test **(C and G)** or Unpaired Student's t test, two tailed **(K, O and S)**: * p <0.05 ,*** p < 0.001. Tumor growth in vivo was assessed for 28 days after the subcutaneous injection of 10 x 10^6^ cells. Graphs in **D**, **L**, **P**, **T**  show the tumor volume and values are expressed as mean ± SD (n=1 independent experiments performed in quadruplicate (D); n=3 indipendent experiments performend in quintuplicate (L, P, T)). One-way ANOVA with Bonferroni test **(D)** or Unpaired Student's t test, two tailed **(L, P and T)**: * p <0.05 ,*** p < 0.001.

**Supplementary Figure S3:** NLGN1 re-expression restores transendothelial migration in vitro**.** HuTu 80and SNU-C2Acontrol (shCTRL) cells or in with NLGN1 is downregulated with two specific short hairpin RNA sequence targeting NLGN1 (shNLGN1b and c),  were further transfected to overexpress a HA-tagged mouse NLGN1 (mNLGN1 HA). **A)** Expression level of human and murine NLGN1 evaluated through qRT-PCR. Fold-change was calculated with respect to control cells. Graph is representative of one experiment performed in triplicate. **B)** Cell lysates were separated using sodium dodecyl sulfate polyacrylamide gel electrophoresis (SDS-PAGE). Immunoblottings were carried out using the rabbit antibody specifically recognizing HA-tag of murine NLGN1 (band detected at 120 kDa), and the mouse monoclonal anti GAPDH antibody (band detected at 37 kDa), as an housekeeping. **C and D)**Transendothelial migration of HuTu 80 **(C)** and SNU-C2A **(D)** cells was recorded in real time through the X-Celligence System for 12 hours.  Histograms show the cell index in terms of percentage relative to start, at the indicated time points. A lower cell index in this representation means an increased capacity of trans-endothelial crossing. Values are expressed as mean ± SD (n=3 independent experiments performed in triplicate). Two-way ANOVA with Bonferroni test: * p < 0.05, ** p < 0.01, *** p < 0.001.

**Supplementary Figure S4:** NLGN1 expression facilitates the adhesion of colorectal cancer cells to endothelial cells in vitro. 4 x 10^4^ HuTu 80 **(A and C)**, SNU-C2A **(B and D)** (shCTRL, shNLGN1b and shNLGN1c) or HCT116 **(E)** and HCT8 **(F)** and HT-29 **(G)** (pLVX and pEZNLGN1) cells, further transduced with a construct expressing the fluorescent protein GFP, were seeded onto a monolayer of HUVECs and allowed to adhere. After 5 hours adherent cells were photographed under a fluorescence microscope and manually counted. Fluorographs in **A and B** are representative of HuTu80 **(A)** and SNU-C2A **(B)** cells attached to HUVECs. Histograms in **C**, **D**, **E**, **F** and **G** show the average cell number and values are expressed as mean ± SD (n=16).  Kruskal-Wallis test with Dunn’s posttest: * p < 0.05, ** p < 0.01, *** p < 0.001.

**Supplementary Figure S5**: A murine orthotopic model was used to assess metastatic invasion in vivo.1,5x 10^6^ cells**(A-D)** HCT116 pLVX and pEZNLGN1 cells and **(E-H)** SNU-C2A cells, further infected with a CMV-Luc vector were orthotopically inoculated into cecum of 6 weeks old NOD/SCID mice. After 8 weeks mice were subcutaneously inoculated with 15 mg/mlluciferine 5 minutes before the sacrifice and the cecum, intestin, stomach, spleen/pancreas, liver and lungs were surgically excised. Luciferine bioluminescence was recorded through IVIS Lumina II apparatus. Images of the liver in **A** and **E** andof the lungs in **C** and **G** metastases are representative of 5 mice. Graphs in **B**, **D**, **F** and **H** show the bioluminescence of tumor cells as total flux. Values are expressed as mean ± SD, n=10. Unpaired Student's t test, two tailed: *, p < 0.05 **, p < 0.01, ***, p < 0.001.

**Supplementary Figure S6:**APC cortical signal quantification. Cortical APC levels were manually identified through the line tool of ImageJ (here indicated by the dashed line). Signal intensity was quantified through the ImageJ Software.

**Supplementary Figure S7:** NLGN1 overexpression localizes APC at the plasma membrane. Confocal microscopy analysis of NLGN1 and APC co-staining in HCT116 **(A and B)**, HCT8 cells **(C and D)** and HT-29 **(E and F)** cells, pLVX and pEZNLGN1. **A, C and E).**Cells were immunostained with anti APC (blue) and anti NLGN1 (red) antibodies. Scale bar: 20 μm. The graphs in **B**, **D** and **F**  show the quantification of APC signal at the cortical area of the cell using the imageJ Software and values are expressed as mean ± SD (HCT116: n=20; HCT8: n=26; HT29: n=34). Mann-Whitney test, two tailed:  ** p < 0.01, *** p < 0.001.

**Supplementary Figure S8:** Co-immunoprecipitation analysis of NLGN1/APC interactions in colorectal cancer cells. Co-immunoprecipitation assays were performed on HuTu 80 **(A and B)**, SNU-C2A **(A and C)**, HT-29, pLVX and pEZNLGN1, **(A and D)**. Proteins (4mg) were immunoprecipitatedeither with the rabbit anti-NLGN (L067) or the rabbit anti-APC antibodies. Western blottings (WB) were carried out using the rabbit anti-NLGN1 antibody (A) specifically recognizing a band detected at 120 kDa and the rabbit anti-APC antibody recognizing bands at 310 kDa (full lenght APC) and at  250 kDa in HuTu 80 (B) and in SNU-C2A (C );  bands at 250 kDa, at 160 kDa and  95-100kDa, which likely corresponds to the MW of the mutant E853* in HT-29 (highlighted with the black asterisks) (D). A clear “gel shifting” is visible for this last band in the samples where APC was immunoprecipitated (**D, right panel**) and blotted vs the samples where NLGN1 was immunoprecipitated (**D, left panel**).  The images are representative of 1 out of 3 reproducible experiments.

**Supplementary Figure S9**: NLGN1 promotes the translocation of CXXC4 to the plasma membrane. Confocal microscopy analysis of NLGN1 and CXXC4 co-staining in HuTu 80 **(A and B)**, shCTRL, shNLGN1b and c, and in HCT116 **(C and D)**, HCT 8 cells **(E and F)** and HT-29 **(G and H)** cells, pLVX and pEZNLGN1. **A, C, E and G)** Cells were immunostained with anti CXXC4 (magenta) and anti NLGN1 (red) antibodies. Scale bar: 20 μm. The graphs in **B**, **D**, **F** and **H**  show the quantification of CXXC4 signal at the cortical area of the cell using the imageJ Software and values are expressed as mean ± SD (HuTu 80: n=18; HCT116: n=6; HCT8: n=19; HT29: n=23 ). Kruskal-Wallis test with Dunn’s posttest **(B)** and Mann-Whitney test, two tailed **(D,F and H)**:  * p < 0.05, ** p < 0.01, *** p < 0.001.

**Supplementary Figure S10:** NLGN1 is specifically involved in β-cat nuclear translocation**.**HuTu 80and SNU-C2Acontrol (shCTRL) cells or in with NLGN1 is downregulated with two specific short hairpin RNA sequence targeting NLGN1 (shNLGN1), were further transfected to overexpress a HA-tagged mouse NLGN1 (mNLGN1 HA) as shown in Supplementary Fig S3.**A-D)** Confocal analysis of nuclear β-cat content. **A and C)** Cells were immunostained with anti α-cat (magenta) and anti β-cat (red) antibodies. Nuclear β-catwas calculated by subtracting the α-cat signal from the β-cat signal quantified by using the ImageJ software. Scale bar: 20 μm. The graphs in **B** and **D**show the fluorescent intensity of nuclear β-catand values are expressed as mean ± SD (HuTu 80: n=35; SNU-C2A: n=45). Mann-Whitney test: *, p < 0.05 **, p < 0.01, ***, p < 0.001.

**Supplementary Figure S11:** NLGN1 promotes a mesenchymal phenotype in colorectal cancer cells. Cell morphology of **(A)**HuTu 80 (shCTRL, shNLGN1b and shNLGN1c) and **(B)** HCT116 (pLVX and pEZNLGN1) cells were observed under a phase-contrast microscope. Cell morphology changed from polygonal (black arrowheads), epithelial structure to spindle-like, mesenchymal structure (white arrowheads) where NLGN1 is expressed. Representative phalloidin staining of **(C)**HuTu 80 (shCTRL, shNLGN1b and shNLGN1c) and **(D)** HCT116 (pLVX and pEZNLGN1) cells. Thick actin filaments (stress fibers)were often detected in NLGN1-expressing cells. **E-H)** Scratch assay of **(E and F)**HuTu 80 (shCTRL, shNLGN1b and shNLGN1c) and **(G and H)** HCT116 (pLVX and pEZNLGN1) cells. Images shown in **E** and **G** are representative of T=0h and 24h. Graphs in **F** and **H** represent the rate of cell migration linear area/hand values are expressed as mean ± SD. Kruskal-Wallis test with Dunn’s posttest **(F)** and Mann-Whitney test, two tailed **(H)**: * p < 0.05, *** p < 0.001.
